# Recovery of Over-Target RuO_2_-Based Thick-Film Resistors by Laser Surface Modification and Dual-Step Trimming: An 8P7R Feasibility Study Toward Multilayer Hybrid Integrated Circuits

**DOI:** 10.3390/mi17101164

**Published:** 2026-10-05

**Authors:** Yuan-Hwei Cheng, Jin-Siang Shaw

**Affiliations:** 1 Graduate Institute of Manufacturing Technology, College of Mechanical and Electrical Engineering, National Taipei University of Technology, Taipei 10608, Taiwan; 2 Department of Mechanical Engineering, National Taipei University of Technology, Taipei 10608, Taiwan; jshaw@ntut.edu.tw; 3 Hypro Enterprise Co., Ltd., Taoyuan City 33463, Taiwan

**Keywords:** laser surface modification, dual-step trimming, resistance reduction, RuO2 thick-film resistor, picosecond laser, MOPA laser, multilayer hybrid integrated circuit, post-fire recovery, TCR, thermal shock

## Abstract

Conventional laser trimming can only increase resistance; consequently, an as-fired RuO_2_ thick-film resistor already above its target cannot normally be recovered. This work evaluates a dual-step route in which a laser surface-modification step first lowers resistance and conventional closed-loop up-trimming on a shared laser trimmer then raises it to target. Commercial 8P7R SIP-8 arrays were used as a controlled vehicle before transfer to high-value multilayer hybrid integrated circuits (HICs). Two complete modification recipes were compared: a picosecond (Pico) route (10–12 ps, 1064 nm) and a nanosecond master-oscillator power-amplifier (MOPA) fiber-laser route (≈6 ns, 1064 nm); because pulse duration, average power, and focusing lens differ simultaneously, the routes are compared as complete recipes rather than as isolated laser-source effects. Across 15 panels (11 Pico and 4 MOPA), 6540 unique physical resistors had valid paired pre- and post-process measurements after 75 records with printing defects were excluded before analysis. In modified Rows 1 and 3, the ratio of modified to as-fired resistance (Rf/Ri) was 0.607±0.015 (n=3192) for the Pico route and 0.831±0.052 (n=1175) for the MOPA route; all Pico resistors, but only 77.6% (912/1175) of MOPA resistors, reached Rf/Ri ≤0.870, the level needed to bring a resistor 15% above target below target before up-trimming. Across seven up-trim kerf geometries (n=34 resistors per cell, two panels per route), the observed Pico–MOPA differences in trimmed ratio across the 15 matched kerf × row cells in which both route means lay within ±1% of target fell within an absolute engineering equivalence margin of ±0.005 in trim ratio (two one-sided tests (TOST)); the scan-cut kept every resistor within ±1%, the I-, D-, and M-cuts had at most 4 of 34 resistors per row outside it, and the U-cut did not hold it; because the cells come from a limited number of panels, this is presented as descriptive engineering evidence rather than population-level proof of route equivalence. After five thermal-shock cycles between −30 and +80 °C, 59/60 modified resistors and all 30 untreated controls remained within ±1%; the modified-minus-control increment was +0.15 percentage points (pp) for Pico (two panels) and +0.09 pp for MOPA (one panel). The MOPA thermal-shock result is presented as preliminary feasibility evidence only. Stage-mean two-point (25/125 °C) temperature coefficient of resistance (TCR) values remained within ±100 ppm °C^−1^ through five processing stages; after 155 °C storage, 2 of 10 MOPA Row 3 resistors exceeded +100 ppm °C^−1^. Top-view scanning electron microscopy (SEM) showed re-solidified surface morphologies without macroscopic delamination, and energy-dispersive X-ray spectroscopy (EDS) detected the principal O, Si, Pb, and Ru signatures; these observations do not establish the conduction mechanism. The contribution is a production-oriented feasibility validation of resistance lowering followed by precision up-trimming for otherwise untrimmable over-target resistors.

## 1. Introduction

Thick-film RuO_2_-based resistors printed on aluminium-oxide (Al_2_O_3_) substrates are a workhorse embedded passive in multilayer hybrid integrated circuits (HICs), where they provide signal-conditioning, pull-up/pull-down, current-sensing, and impedance-matching functions integrated directly into the ceramic stack. The active layer is a screen-printed RuO_2_–PbO–SiO_2_ paste that is co-fired at approximately 850 °C in a belt furnace, producing a percolation network of RuO_2_ grains embedded in a lead-borosilicate glass matrix [1,2,3,4,5,6]. Because a multilayer HIC carries a high per-unit cost—dominated by its co-fired ceramic layers, thin-film metallisation, and downstream assembly—a single out-of-tolerance embedded resistor can force the scrapping of the entire assembly, making post-fire yield recovery a critical manufacturing step.

In practice, the as-fired resistance value of such a thick-film resistor deviates from its design target by 3–8% on average, and by up to 15% on individual panels, even when the printing screen, paste composition, firing profile, and substrate lot are all held nominally constant [6,7,8]. The origin of this scatter is a combination of print-thickness variation, glass-flow non-uniformity during firing, and lot-to-lot drift of the paste itself; correcting the paste formulation or re-firing is neither economical nor practical for volume production. Because thick-film resistors inside a multilayer HIC are typically specified to a tolerance of ±1% or tighter, the post-fire scatter can cause substantial yield loss and can dominate the cost of nonconforming high-value HIC assemblies. The magnitude is process- and product-specific and should not be interpreted as a universal industry scrapping rate.

Conventional laser up-trimming is the standard industry solution. A focused laser removes a controlled fraction of the conductive layer along a designed kerf pattern (I-cut, L-cut, or serpentine), raising the resistance in defined increments until the target value is reached, at which point the trim is stopped in real time by an in-line four-wire measurement [9,10,11,12,13,14,15]. Two families of pulsed laser sources are relevant to the present work:

- **Picosecond (Pico) diode-pumped solid-state lasers**, with pulse durations of the order of 10 ps, in which the pulse is short compared with the thermal-diffusion time of the irradiated layer, so that heat input to the surrounding material is limited [16,17,18,19,20].
- **Master-oscillator power-amplifier (MOPA) fiber lasers**, whose pulse duration is adjustable in the nanosecond range and whose interaction with oxide and glass-containing films is dominated by heating and melting [21,22,23,24].

In the present work, both sources are used only for the resistance-lowering surface-modification step; all conventional up-trim kerfs are cut on a single shared laser trimmer (Section 2.2).

This conventional method has a fundamental one-way limitation: it can correct a resistor below target, but it cannot rescue an as-fired resistor that is already above target. Laser-induced resistance reduction in RuO_2_–glass thick films is not newly discovered here. Gofuku, Ogama, and Takasago reported in 1989 that pulsed Nd:YAG surface modification could markedly decrease resistivity; from morphology, X-ray diffraction (XRD), and X-ray photoelectron spectroscopy (XPS), they proposed that rapid heating and cooling modified the glass and introduced Ru-related electronic carriers without producing a detectable new crystalline Ru–O phase [25]. The present study uses that established phenomenon as the first step of a manufacturing recovery sequence: (1) lower an over-target resistance by laser surface modification, and then (2) apply ordinary feedback-controlled up-trimming to return it precisely to target. The novelty claimed here is the production-oriented validation of this dual-step recovery route—including process latitude, trim precision, thermal shock, TCR ageing, and within-panel controls—rather than discovery of the resistance-lowering phenomenon itself.

The two recipes have similar nominal pulse energies (about 10 and 14 μJ) but pulse durations of 10–12 ps and ≈6 ns, so their estimated peak powers differ by roughly 400 times (≈0.9 MW versus ≈2.3 kW); peak intensity and fluence cannot be compared because the focused spot size was not measured. It is, therefore, not self-evident that both routes should produce equivalent product-level performance, and, to the knowledge of the authors, no single study has compared them on the same thick-film resistor geometry using an experimental vehicle that provides a co-fired within-panel untreated control row against which the laser-induced increment can be resolved.

Before a laser-trim recovery methodology can be committed to production HIC panels—where each mis-processed substrate destroys a high-value multilayer assembly—its transferability, precision, and post-modification reliability must first be verified on a controlled experimental vehicle. To that end, the present work uses **commercial 8P7R SIP-8 resistor arrays**—8-pin single-in-line packages each containing seven co-fired RuO_2_ thick-film resistors sharing a common bussed pin—as the test vehicle. The 8P7R array was chosen because (i) it is fabricated by the same screen-print, co-fire, and laser-trim process used for embedded resistors inside HICs, (ii) its regular geometry provides a co-fired resistor set with an untreated within-panel control row, and (iii) it is independent of downstream HIC assembly cost, allowing the laser-trim behaviour itself to be isolated. The intent is that a route verified on the 8P7R vehicle can subsequently be re-evaluated on the embedded RuO_2_ resistors of high-value multilayer HIC substrates.

We manufactured 17 nominally identical 8P7R panels from a single alumina lot: 11 panels were allocated to Pico laser modification, 4 to MOPA laser modification, and 2 to an untreated control (panel identifiers 2–9 and *b*–*d* for Pico, *e*–*h* for MOPA, and *j*–*k* for control). We then evaluated the finished components against four production-related metrics:

1. **Downward recovery headroom**—the ratio Rf/Ri after the resistance-lowering surface-modification step, followed by verification that conventional up-trimming can reach the ±1% customer window.
2. **Up-trim precision**—the run-to-run standard deviation of the trimmed resistance at 25%, 50%, and 75% increment, evaluated for seven kerf geometries (I-cut, D-cut, M-cut, L-cut with sharp corner, L-cut with rounded corner, U-cut, and scan-cut) cut on the shared trimmer.
3. **Thermal-shock response, compared against a within-panel untreated control**—the percentage resistance change ΔR/R after five thermal-shock cycles between −30 °C and +80 °C, measured on laser-modified rows and on a co-fired untreated row of the same 8P7R panel, so the increment attributable to laser modification is separated from the baseline drift of this 8P7R lot.
4. **Long-term TCR stability**—the evolution of the temperature coefficient of resistance through five sequential production stages (post-fire → post-laser → post-trim → post-thermal-shock → post-155 °C × 100 h high-temperature storage).

The remainder of the paper is organised as follows. Section 2 describes the resistor geometry, laser process parameters, panel allocation, and statistical framework. Section 3 reports the four production-related metrics in the same order given above and provides surface-characterisation evidence (SEM + EDS) concerning morphology and retention of the principal elemental constituents after processing. Section 4 discusses the practical implications of the results for manufacturers who must choose between Pico and MOPA equipment. Section 5 summarises the conclusions.

**Figure 1:**
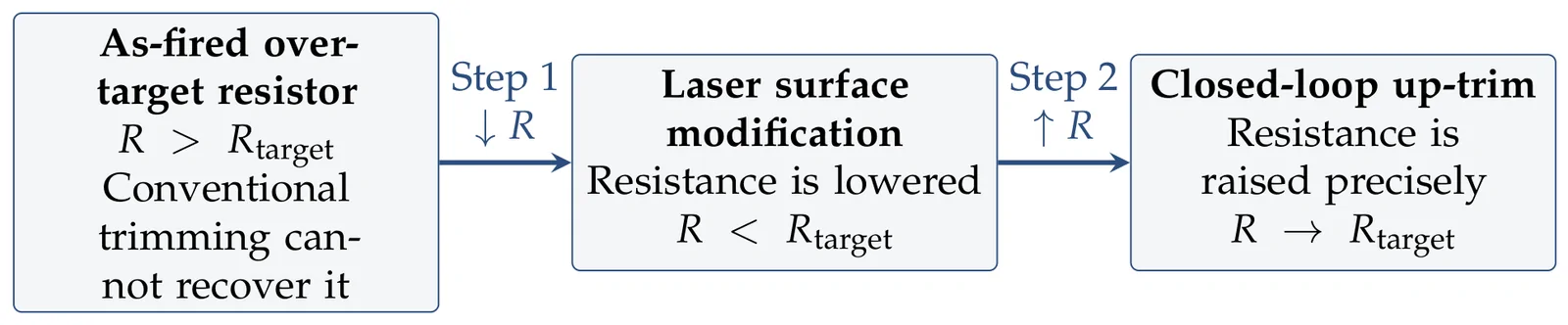
Dual-step recovery concept for an over-target thick-film resistor. The resistance-lowering surface-modification step creates adjustment headroom; conventional feedback-controlled trimming then raises the resistance to the final target.

Figure 1 states the central process logic. The first laser operation is deliberately not a conventional trim cut: it creates downward resistance headroom. Only after the value has crossed below the target is the established closed-loop up-trim operation used for final precision.

## 2. Materials and Methods

### 2.1. Experimental Vehicle—8P7R Resistor Array

All resistors reported in this study are the co-fired thick-film elements of a **commercial 8P7R SIP-8 resistor array**—an 8-pin single-in-line package containing **seven RuO_2_ thick-film resistors sharing a common bussed pin**—printed on an alumina (Al_2_O_3_, 96%) substrate of 0.635 mm thickness. The specific resistance value of the array is not material to this study (the laser-trim behaviour scales with kerf geometry and RuO_2_/glass microstructure, not with the nominal resistance); the 8P7R array is used strictly as an experimental vehicle whose regular, co-fired geometry allows a large sample of nominally identical thick-film resistors to be evaluated under identical print-and-fire conditions with within-panel controls.

Each panel is manufactured as an alumina test substrate carrying **three parallel rows of the 8P7R geometry, each row containing multiple individually-addressable resistors**:

- **Row 1 (Row1_perpendicular)**—the laser-modified row, with the surface-modification scan lines oriented perpendicular to the current-flow direction.
- **Row 2 (Row2_untreated)**—a within-panel untreated reference row that serves as the internal control for every subsequent reliability measurement.
- **Row 3 (Row3_parallel)**—a second laser-modified row, with the surface-modification scan lines oriented parallel to the current-flow direction.

The 3-row layout was chosen so that every laser measurement is paired with a within-panel untreated reference; this removes batch-to-batch paste and firing variability from the reliability statistics and is the origin of the **Row 2 control column** that appears in all subsequent figures and tables.

The active layer is a commercial RuO_2_/PbO–SiO_2_ paste (thick-film resistor paste, sheet resistance nominal 100 Ω sq^−1^) applied by 325- mesh stainless-steel screen and fired at 850 °C/10 min peak plateau in an inline belt furnace. Silver–palladium termination electrodes are pre-fired at 850 °C and the resistor layer is over- printed and co-fired in a second pass. The as-fired resistance distribution is measured at room temperature (approximately 25 °C) using an Agilent 34410A 6$\frac{1}{2}$-digit digital multimeter (Agilent Technologies, Santa Clara, CA, USA).

### 2.2. Laser Modification Systems

The dual-step sequence uses two different laser operations, which are performed on different equipment and must not be confused (Figure 1):

- **Step 1—surface modification (resistance lowering).** Either the Pico or the MOPA source raster-scans the *whole* surface of each resistor in the modified row. No kerf is cut in this step; the beam is scanned in parallel lines at a fixed line spacing (hatch) of 0.01 mm, with a single pass, over the entire resistor area. The scan lines are perpendicular to the current path in Row 1 and parallel to it in Row 3. The modified resistance Rf is measured after this step and before any up-trim.
- **Step 2—conventional up-trim (resistance raising).** A single laser trimmer, shared by all panels irrespective of the modification route, cuts a closed-loop kerf (I-, D-, M-, L-, U- or scan-cut) until the target resistance is reached.

**Pico modification system.** Photonics Industries RGH-1064-5 picosecond laser (Photonics Industries International, Inc., Ronkonkoma, NY, USA), operated at the following settings for all Pico panels:

- Wavelength: 1064 nm; pulse duration: 10–12 ps;
- Repetition rate: 100 kHz; average output power: 1 W, i.e., a nominal pulse energy of $E_{\mathrm{pulse}}=P_{\mathrm{avg}}/f_{\mathrm{rep}}\approx 10\;\mu$J;
- Scan speed: 2000 mm s^−1^; one pass; hatch 0.01 mm; F100 focusing (f-theta) lens.

**MOPA modification system.** JPT YDFLP-20-M7 MOPA fiber laser (Shenzhen JPT Opto-electronics Co., Ltd., Shenzhen, China), operated at the following settings for all MOPA panels:

- Wavelength: 1064 nm; pulse duration: ≈6 ns;
- Repetition rate: 100 kHz; average output power: 1.4 W, i.e., a nominal pulse energy of ≈14μJ;
- Scan speed: 2000 mm s^−1^; one pass; hatch 0.01 mm; F160 focusing (f-theta) lens.

With a scan speed of 2000 mm s^−1^ at 100 kHz, the nominal pulse-to-pulse pitch along each scan line is 20 μm for both routes, and the line-to-line pitch is 10 μm. The focused spot diameter, fluence, and the depth of the modified layer were not measured, and no fluence-based argument is used in this paper.

**Shared up-trim laser trimmer and kerf dimensions**. All up-trim kerfs in Section 3.2 and Section 3.3 were cut on the same laser trimmer, with the same closed-loop resistance feedback, for both routes: an ESI 4990 laser trimming system (Electro Scientific Industries, Beaverton, OR, USA) with a diode-pumped Nd:YAG laser source (1064 nm). The cutting parameters (e.g., power, repetition rate, and cut speed) were not contained in the archived experiment record and are therefore not reported; because both routes used the same trimmer, these settings are common to the Pico–MOPA comparison. The kerf geometries (I-cut, D-cut, M-cut, L-cut with sharp corner, L-cut with rounded corner, U-cut, and scan-cut) are shown in Supplementary Figure S2. The kerf width was measured at two sites with the trimmer inspection microscope and a two-axis FUJI linear scale as 0.042 and 0.043 mm (Supplementary Figure S1 and Table S1). The up-trim cut is intended to penetrate the full printed resistor-film thickness to the alumina substrate; the nominal fired-film thickness is approximately 10 μm. The kerf depth was not independently measured by cross-sectional microscopy, so 10 μm is a nominal film thickness and not a measured kerf depth. The kerf length is not a preset parameter: in closed-loop trimming, the cut advances until the measured resistance reaches the target, so the length varies from resistor to resistor and was not logged individually.

**What the recovery comparison can and cannot attribute.** Because Rf is measured after Step 1 and before Step 2, the Rf/Ri results of Section 3.1 are not affected by the up-trim kerf geometry. They are, however, affected by the complete modification recipe: pulse duration (10–12 ps vs. ≈6 ns), average power (1 W vs. 1.4 W), and focusing lens (F100 vs. F160) differ simultaneously between the two routes. The Pico–MOPA recovery difference is therefore attributed to the two complete recipes, not to pulse duration alone. Conversely, because Step 2 is performed on one shared trimmer, the up-trim precision comparison is not confounded by different trimming hardware.

**How the resistance decrease was obtained.** Operationally, the resistance decrease is produced by Step 1 alone: the whole resistor surface is raster-scanned once with the parameters listed above, and the resistance of every resistor is re-measured immediately afterwards (Rf). No material is intentionally removed and no kerf is cut in this step. The present experiments quantify the electrical outcome of this step; they do not isolate its microscopic mechanism. Thus, in this work, “resistance-lowering laser modification” refers specifically to the experimentally observed decrease in resistance after one full-surface raster scan under the stated processing conditions, before any trimming kerf is introduced. A possible interpretation, based on the literature rather than on measurements made here, is the laser-induced modification of the Ru-containing glass phase reported by Gofuku et al. [25] (Section 3.5).

### 2.3. Panel Allocation and Processing Traceability

Seventeen panels were manufactured from a single alumina lot and a single resistor-paste batch, and were allocated as summarised in Table 1. The allocation is reproduced verbatim from the fabrication laboratory-notebook record to make sample tracking fully transparent for the reader.

**Thermal-shock panel allocation—explicit acknowledgement.** As indicated in row 9 of Table 1, panel *g*—one of the three panels allocated to the initial thermal-shock experiment—was excluded from analysis because Row 1 of that panel exhibited anomalous initial resistance values that were traced back to a mis-registered kerf and could not be reconciled with the remaining resistors on the panel. A registration error changes where the kerf intersects the current path and therefore changes the effective remaining conductive cross-section; the resulting resistance shift represents a process-placement failure rather than the intended laser treatment. The initial Pico thermal-shock run was likewise incomplete, and two further Pico panels from the original 11 (#8 and #9) were thermal-shock tested in a make-up run on 10 November 2025 to complete the paired-ΔR/R Pico dataset (row 10 of Table 1). A subsequent MOPA make-up run was executed on panel *e* (row 11 of Table 1) to add MOPA thermal-shock data to the study; the pre- and post-shock resistance sessions on panel *e* were re-tabulated with matched resistor-position labels across all three rows and all five resistor positions per R-group, so per-resistor ΔR/R is paired position-by-position within the same panel-row on Pico panels #8 and #9 and on MOPA panel *e*. Section 3.3 therefore reports thermal-shock reliability as a per-resistor paired ΔR/R across all three rows of the three thermal-shock panels (*n* = 90 individual resistors: 60 Pico + 30 MOPA), and as the difference between the post-trim and post-thermal-shock TCR stage means (for Pico these two stages were measured on different panels; see Section 3.4). The remaining comparisons in Section 3.1, Section 3.2, Section 3.4 and Section 3.5 use the full Pico + MOPA panel set.

**Resistance-recovery data inclusion.** Each of the 15 laser-route panels contains 3×7×21=441 unique physical resistors, for 6615 possible paired pre- and post-process observations (Pico 4851; MOPA 1764). Seventy-five records (71 Pico and 4 MOPA) were excluded because the resistor had a printing defect, identified from its as-fired value before laser processing. The exclusion was therefore applied before calculation of Rf/Ri and was not based on the magnitude or direction of the recovery outcome. This left 6540 unique paired observations (4780 Pico and 1760 MOPA), one observation per physical resistor. The Rf/Ri recovery analysis uses only the laser-modified Rows 1 and 3 (Pico n=3192; MOPA n=1175); the untreated Row 2 is reported separately (Pico n=1588; MOPA n=585). The panel-level exclusions of Table 1 (e.g., panel *g* in the thermal-shock allocation) are separate from, and in addition to, this record-level screen.

### 2.4. Measurement Procedures

The instruments and conditions below are those recorded in the laboratory instrument list for this study.

**Resistance measurement.** All resistance values were measured at room temperature (approximately 25 °C) with an Agilent 34410A 6$\frac{1}{2}$-digit digital multimeter. Every resistor on every panel was measured, giving the resistance-recovery sample sizes reported in the preceding data-inclusion paragraph. Only complete pre/post pairs were analysed.

**TCR measurement.** The temperature coefficient of resistance was determined by a two-point method comprising four steps: (1) $R_{1}$ was measured at room temperature (25 °C); (2) a FONTON SMD-928 hot-plate station was set to 125 °C and allowed to stabilise for about 10 min; (3) the resistor array was placed flat on the hot plate for 3 min and $R_{2}$ was then measured with the same multimeter; and (4) TCR calculated from TCR (ppm °C^−1^) $=10^{6}\times \left(R_{2}-R_{1}\right)/\left[R_{1}\times \left(125-25\right)\right]$. This is the hot-TCR convention used for acceptance testing of thick-film resistors against a ±100 ppm °C^−1^ specification; the two-point value is a chord slope over 25–125 °C and does not describe any curvature of R(T) between the end points. Reported values are the arithmetic mean and standard deviation across the sampled resistors of each row and stage. At the post-fire stage, four resistors per row were sampled from each route panel (n=44 per Pico row, n=16 per MOPA row). At the post-laser stage, 14 resistors per row were measured on each of the 11 Pico panels and the 4 MOPA panels listed in Table 1, giving n=154 per Pico row and n=56 per MOPA row. Later reliability stages used the stage-specific sample sizes reported with the results. Each resistor was measured once per stage: no repeat measurements and no intermediate temperatures were recorded, so a measurement-repeatability estimate cannot be given from these data. The reported standard deviations therefore combine resistor-to-resistor variation with measurement variation, and differences of a few ppm °C^−1^ between stages should not be over-interpreted (see the limitations in Section 5).

**Thermal-shock test.** Panels were exposed to five cycles between +80 °C and −30 °C in a thermal-shock tester (KSON TSK-A3T-150; KSON, New Taipei City, Taiwan). International Electrotechnical Commission (IEC) standards IEC 60068-2-14 [26] and IEC 60115-1 [27] were used only as general change-of-temperature and fixed-resistor reliability frameworks, and MIL-PRF-55342 [28] only as a reference for film-resistor qualification practice; the five-cycle −30/+80 °C exposure is a short screening condition chosen by the manufacturer and is milder than typical qualification severities, so no IEC conformity is claimed. Resistance was measured at room temperature before the first cycle and after the fifth cycle. The percentage change ΔR/R was computed on a per-resistor basis; 10 individual resistors per row on each panel were tracked (thus 20 per row across the two Pico panels and 10 per row on the single MOPA panel).

**High-temperature storage test.** Panels were placed in a hot-air circulation oven (H150; ACE Dragon Corp., Hsinchu County, Taiwan) at 155 °C for 100 h as an accelerated screening condition. The results were interpreted with reference to the general stability framework of IEC 60115-1 [27]; the acceptance criteria used here are the manufacturer’s study criteria and are not claimed as IEC qualification. Resistance and TCR were re-measured at room temperature after cool-down.

**SEM and EDS characterisation.** Surface morphology was imaged on a Zeiss field-emission scanning electron microscope operating at an electron high tension (EHT, accelerating voltage) of 10 kV with the chamber secondary-electron detector (SE2), at 1 kX magnification; a working distance of 8.7–8.8 mm and 60 μm aperture were used throughout. EDS spectra were recorded and interpreted following the semi-quantitative light-element methodology described by Newbury and Ritchie for silicon-drift-detector (SDD) EDS of oxide systems [29,30]. Energy-dispersive X-ray spectra were acquired on the same instrument using an Oxford X-Max SDD (Oxford Instruments, Abingdon, UK) at EHT = 15 kV with LIVETIME = 44–66 s per spectrum. All specimens were sputter-coated with approximately 5 nm of gold before imaging to eliminate charging.

### 2.5. Statistical Framework

All statistical comparisons reported in this work use the following convention:

- Continuous outcomes (resistance change percentage, TCR value, trimmed-resistance standard deviation) are reported as mean ± standard deviation with the sample size *n* explicitly stated for every comparison.
- **Statistical unit and independent replicates.** The unit of observation is the individual physical resistor, measured once per stage. Resistors are nested in panels, and the panel is the independent replicate of the processing route. The number of panels therefore limits inference: resistance recovery uses 11 Pico and 4 MOPA panels; up-trim precision uses one panel row per cell (Pico panels #2 and #5, MOPA panels *e* and *f*; n=34 interior resistors per cell, 17 from each panel); thermal shock uses two Pico panels (#8, #9) and one MOPA panel (*e*), with 10 resistors per row; TCR uses the sample sizes given in Section 2.4.
- With at most two panels per route in each test (thermal shock: two Pico panels and one MOPA panel; up-trim: two panels per route), a mixed-effects model with a panel random effect cannot be estimated reliably (the between-panel variance would rest on at most one degree of freedom). Resistor-level intervals are therefore reported as descriptive of the tested panels, and a panel-level analysis is added wherever at least two panels per route exist (thermal shock, Pico).
- The up-trim comparison between Pico and MOPA uses matched (kerf × row) cells as the analysis unit. A cell enters the primary comparison only if the cell mean of both routes lies within ±1% of the target ratio; this rule, which follows the product tolerance, retains 15 of the 21 cells (it excludes the U-cut at all three increments and the L-cut with sharp corner at +50% and +75% and with rounded corner at +75%). The earlier 17-cell set (all cells except the L-cuts at +75% and the U-cut at +50% and +75%), the 14 cells of the combinations that held the window (scan-, I-, D-, and M-cuts, and the L-cuts at +25%), and all 21 cells are reported as sensitivity analyses; these cells are not independent panel replicates, so the result is descriptive of the tested panels. The comparison applies a paired two-one-sided-tests (TOST) equivalence procedure with an engineering-defined margin; Welch’s unequal-variance *t*-test (α = 0.05) is used for descriptive two-group contrasts. The equivalence margins (±0.005 absolute in trim ratio, i.e., 0.29–0.40% of the 1.25–1.75 targets, and ±0.5 pp in thermal-shock ΔR/R, i.e., one half of the ±1% customer window) were fixed from the customer tolerance before the equivalence tests were run, but were not pre-registered before data collection.
- The thermal-shock effect of laser modification (Section 3.3) is presented as a **descriptive within-panel contrast** between laser-modified rows (Rows 1 and 3) and the co-fired untreated row (Row 2) on the same 8P7R panel. The design intent is to isolate the increment that laser modification adds on top of the baseline thermal-shock drift of the 8P7R lot itself; per-resistor ΔR/R is paired position-by-position within each panel-row.
- Individual resistor readings are nested within panels and are not independent panel replicates. Resistor-level statistics are therefore descriptive of the tested panels. Panel counts are reported alongside resistor counts, and inference beyond the tested panels is avoided, especially for the MOPA thermal-shock result obtained from one panel. A larger multi-panel study or a pre-specified mixed-effects analysis is required for population-level comparison of production routes.
- Computations were performed in Python 3.12 using SciPy 1.14 and pandas 2.2. Analysis code and processed data are available from the corresponding author upon reasonable request.

## 3. Results

The four production-related performance metrics defined in the Introduction are reported here in the same order, followed by a compact direct-material verification (Section 3.5).

### 3.1. Resistance Recovery from Post-Fire Out-of-Tolerance

**Figure 2:**
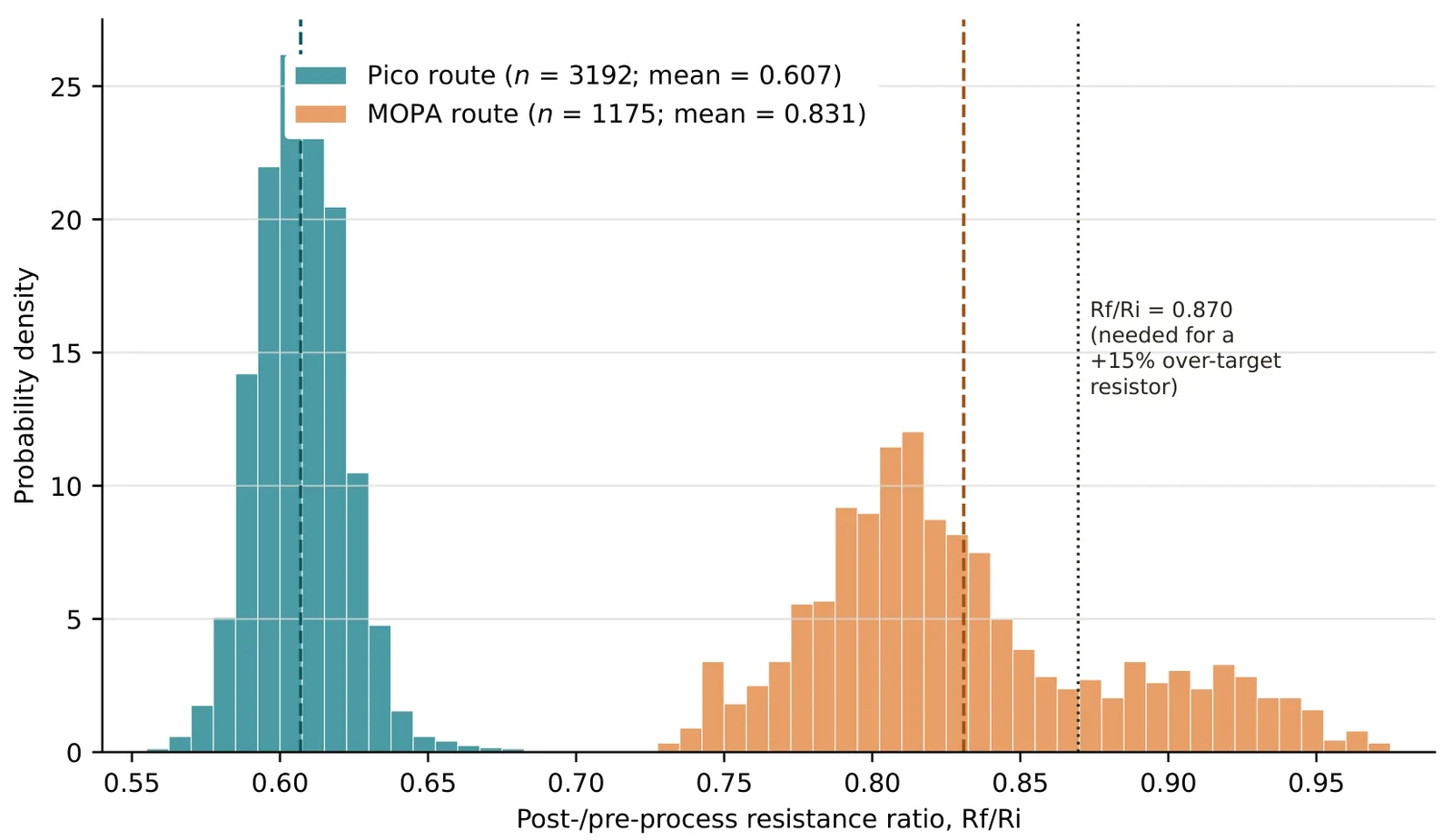
Distribution of the post-laser resistance-recovery ratio Rf/Ri for Pico surface modification (n=3192 unique resistors, 11 panels) and MOPA surface modification (n=1175 unique resistors, 4 panels). The dotted line marks Rf/Ri =1/1.15=0.870, the ratio needed to bring a resistor that is 15% above target below target. The observed distributions do not overlap; Row 2 within-panel untreated controls sit at Rf/Ri =1.00 and are omitted from the histogram for clarity.

The immediate purpose of laser modification is to bring an over-target as-fired resistance below its target value, so that the subsequent up-trim (Section 3.2) can raise it into the customer tolerance window. Figure 2 reports the post-laser resistance-recovery ratio Rf/Ri (the ratio of the modified resistance to the original as-fired resistance) aggregated across the 11 Pico-modified and 4 MOPA-modified panels of Table 1. Rf is measured after the surface-modification step (Step 1) and before any up-trim.

The two modification recipes produce clearly separated distributions of per-resistor Rf/Ri values (each panel row carries three 8P7R SIP-8 arrays, and each array contributes seven individually-addressable resistors, for a total of 21 measurements per panel row):

- **Pico surface modification** delivers Rf/Ri = **0.607 ± 0.015** across *n* = 3192 unique resistors (Row 1 perpendicular + Row 3 parallel, aggregated over the 11 Pico panels of Table 1), with an inter-resistor coefficient of variation of 2.5%.
- **MOPA surface modification** delivers Rf/Ri = **0.831 ± 0.052** across *n* = 1175 unique resistors (Rows 1 and 3 of the 4 MOPA panels of Table 1), with a coefficient of variation of 6.2%.

The observed ranges are separated (Pico 0.551–0.681; MOPA 0.729–0.975). Practically, Pico modification reaches a resistance value that is approximately 39% below the as-fired reading, whereas MOPA modification reaches 17% below. Because pulse duration, average power, and focusing lens differ simultaneously between the two recipes (Section 2.2), the difference is attributed to the complete Pico and MOPA modification recipes rather than to pulse duration alone. The up-trim kerf geometry plays no role in this comparison, because Rf is measured before up-trim. A resistor that is 15% above target must reach Rf/Ri ≤1/1.15=0.870 to be brought below target before up-trim. All 3192 Pico-modified resistors met this condition (maximum Rf/Ri 0.681). For the MOPA recipe, 912 of 1175 resistors (77.6%) reached Rf/Ri ≤0.870; the remaining 22.4% would cover only a smaller over-target deviation in a single pass (the largest observed ratio, 0.975, corresponds to about +2.6%). The mean MOPA ratio (0.831) corresponds to about +20% coverage, but the per-resistor tail, not the mean, determines whether a given over-target resistor can be recovered under the tested MOPA recipe. Table 2 lists the panel-level values: the MOPA panel means range from 0.804 to 0.843 while the within-panel SDs are 0.047–0.051, so the broad MOPA distribution arises mainly within panels rather than between them. The resistance-lowering step itself does not place a resistor inside the ±1% window; it moves the resistor below target, and the final tolerance is set by the subsequent up-trim (Section 3.2).

A factorial experiment varying pulse duration, power, and lens independently would be required to attribute the 39% versus 17% shifts to any single laser parameter.

Two secondary features of Figure 2 also deserve mention. First, the Pico route produced a narrower ratio distribution than the MOPA route (SD 0.015 vs. 0.052). Because several recipe parameters differ simultaneously, the origin of that difference cannot be isolated from these data. Second, no Pico or MOPA resistor exceeded Rf = Ri (i.e., no event with Rf/Ri >1 was observed among the 4367 laser-modified resistors with valid paired measurements). The within-panel Row 2 untreated controls, aggregated across the same panels, return Rf/Ri = 1.000 ± 0.013 (Pico controls, *n* = 1588) and 1.000 ± 0.001 (MOPA controls, *n* = 585, i.e., the within-panel Row 2 of the same MOPA panels), confirming that no collateral drift is induced in adjacent unmodified resistors on the same substrate.

### 3.2. Up-Trim Precision at 25%, 50%, and 75% Increment

**Figure 3:**
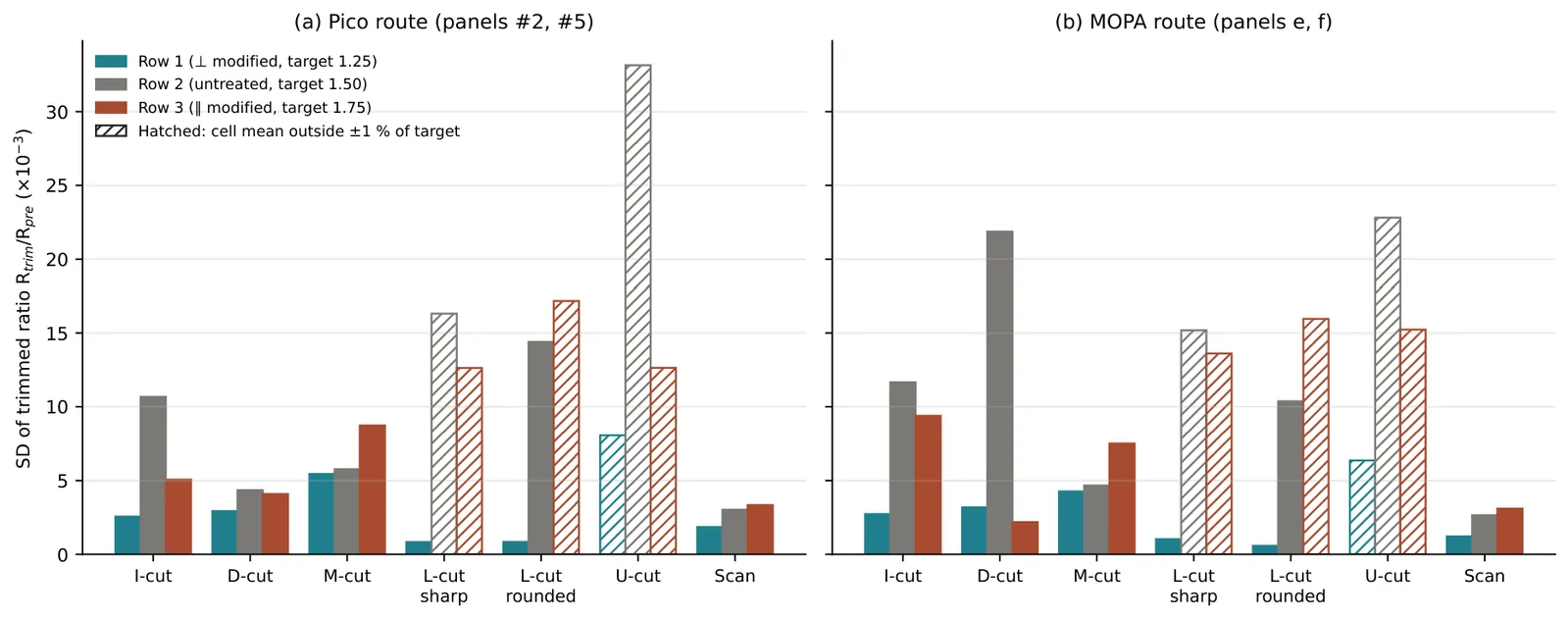
Up-trim precision on the shared trimmer, expressed as the standard deviation of the trimmed ratio (post-trim/pre-trim resistance) for each kerf geometry and row, for (**a**) the Pico route and (**b**) the MOPA route (n=34 resistors per bar from two panels; MOPA U-cut Row 1: n=17, panel *f* only). Hatched bars: cell mean outside ±1% of target. Values are listed in Table 3.

Once the modification step has brought the resistance below target, the resistor is subjected to closed-loop up-trim to the final target value on the shared trimmer. Each route was exercised across the same seven kerf geometries—**I-cut, D-cut, M-cut, L-cut with sharp corner, L-cut with rounded corner, U-cut, and scan-cut** (Supplementary Figure S2). Each row of a panel is trimmed to a single row-specific target ratio (post-trim/pre-trim resistance): Row 1 (modified, scan perpendicular to the current path) to 1.25 (+25%), Row 2 (untreated) to 1.50 (+50%), and Row 3 (modified, scan parallel) to 1.75 (+75%). The panels used for this matrix are Pico panels #2 and #5 and MOPA panels *e* and *f*. Each cell pools the corresponding row of the two panels of a route (n=34 resistors, 17 per panel); the four edge positions (1, 2, 20, 21) of each row were excluded beforehand because routine panel inspection flags them as edge-defect-prone. Table 3, generated directly from the per-panel measurement records (per-panel values in Table S2 of the Supplementary Materials), gives *n*, mean, SD, bias, and the number of resistors outside ±1% of target for every laser × kerf × row cell, and Figure 3 shows the SDs.

**Equivalence of the two routes.** The primary comparison uses the 15 kerf × row cells in which the cell mean of both routes lies within ±1% of target (Section 2.5). Using the cell as the unit, the paired Pico − MOPA difference in mean trimmed ratio is $+1.6\times 10^{-3}$; its 90% confidence interval, $\left[+0.9\times 10^{-3},\;+2.2\times 10^{-3}\right]$, lies inside the equivalence margin of $\pm 5\times 10^{-3}$ (an absolute margin in trim ratio, corresponding to 0.29–0.40% of the 1.25–1.75 targets), and the paired TOST gives p<0.001. The Pico mean was slightly higher in 14 of the 15 cells, i.e., there is a small systematic offset of about 0.1% of target, well inside the margin. For the precision itself, the Pico/MOPA SD ratio over the same 15 cells has a geometric mean of 0.99 (range 0.20–1.87; 90% confidence interval 0.78–1.27), which lies inside an equivalence band of 0.5–2 for the SD ratio (TOST on log SD ratio, p<0.001). Three sensitivity analyses were run. With the earlier 17-cell set (which also contains the L-cut with sharp corner at +50% and the U-cut at +25%, whose means lie outside ±1%), the difference is $+1.7\times 10^{-3}$$\left[+1.1,\;+2.4\right]\times 10^{-3}$ (p<0.001). Restricting the comparison to the combinations that held the window (scan-, I-, D-, and M-cuts, and the L-cuts at +25%; 14 cells), it is $+1.5\times 10^{-3}$$\left[+0.8,\;+2.1\right]\times 10^{-3}$ (p<0.001). Including all 21 cells, i.e., also the combinations that did not reach the target, it is $+4.5\times 10^{-3}$$\left[+2.1,\;+6.8\right]\times 10^{-3}$, which extends beyond the margin (TOST p=0.34): in those cells, both routes fell short of the target and the MOPA ratios fell further short (differences of 1.2–$2.3\times 10^{-2}$). The equivalence therefore applies only to the kerf and increment combinations that reached the target.

Two qualifications apply. First, each route contributes two panels, so panel-to-panel variation is not separated from route differences. Second, conformance to the ±1% window was checked resistor by resistor. The scan-cut kept all resistors within ±1% of target in every row. The I-, D-, and M-cuts had 0–4 of 34 resistors per row outside ±1% (at most 3 of 17 in any single panel row). The L-cuts kept all resistors within ±1% at +25%, had 10–24 of 34 outside at +50%, and did not reach the +75% target (cell means 1.64–1.68 instead of 1.75). The U-cut had 30–34 of 34 resistors outside ±1% in every row (MOPA Row 1: 16 of 17). The Row 1 U-cut record of panel *e* contains ratios below 1; it was treated as a recording error and is excluded from Table 3 and from all statistics. The U-cut is therefore not suitable for production use under the tested conditions, and the L-cuts are limited to small increments. The equivalence result concerns mean trimmed ratios and does not imply that every kerf geometry meets the ±1% window. Because the cells are derived from a limited number of panels and are not independent panel replicates, these TOST results are interpreted as descriptive engineering evidence within the tested panels rather than as population-level proof of route equivalence. Because both routes are trimmed on the same trimmer with the same closed-loop feedback, this equivalence is best read as showing that the preceding surface modification, by either recipe, does not measurably disturb the subsequent up-trim.

### 3.3. Thermal-Shock Response—Within-Panel Comparison of Laser-Modified Rows Against the Same-Panel Untreated Row

**Study design**. For each 8P7R panel used in this section, the three resistor rows fabricated in a single print-and-fire cycle are allocated to three treatments before the five-cycle thermal-shock exposure (−30 °C ↔ +80 °C; Section 2.4):

- **Row 1**—surface modification with scan lines perpendicular to the current path (laser-modified);
- **Row 2**—no laser modification (**within-panel untreated control**, same paste, same firing, same package);
- **Row 3**—surface modification with scan lines parallel to the current path (laser-modified).

Because all three rows share a single co-fired resistor lot, package, lead-frame, and shock exposure, the ΔR/R difference between Row 2 and Rows 1 or 3 isolates the effect that laser modification adds on top of the baseline thermal-shock response of this 8P7R lot. This is the reliability question the study is designed to answer: **does laser modification degrade the thermal-shock behaviour of a resistor relative to an untreated resistor sharing the same substrate?**

**Sample coverage.** Post-modification, post-trim, pre- and post-shock resistance was measured at 25 °C on the same five positions of each R-group of each row of each panel, on two Pico panels (#8, #9; make-up run of 10 November 2025) and one MOPA panel (*e*; MOPA make-up run), all taken from the original panel set of Table 1. Per-resistor $\Delta R/R=\left(R_{25,\mathrm{post}}-R_{25,\mathrm{pre}}\right)/R_{25,\mathrm{pre}}$ is therefore paired position-by-position within the same panel-row between the pre-shock and post-shock measurements. The total sample is *n* = 90 individual resistors (60 Pico + 30 MOPA), of which 60 are laser-modified (40 Pico + 20 MOPA) and 30 are within-panel untreated controls (20 Pico + 10 MOPA). Table 4 lists ΔR/R per panel and row, and Table 5 summarises the within-panel comparison of laser-modified rows against the untreated row.

**Interpretation.** On the two tested Pico panels, laser-modified rows drifted 0.15 pp more than the within-panel untreated rows; all 40 modified and 20 untreated resistors remained inside the ±1% study window. On the single MOPA make-up panel, the descriptive modified-minus-control contrast was +0.09 pp; 19/20 modified and 10/10 untreated resistors remained inside the window. Because resistors are nested within only two Pico panels and one MOPA panel, no population-level inferential claim is made from these resistor-level contrasts. The post-shock resistances were recorded with 1 Ω resolution, i.e., 0.05–0.11% of the 0.93–1.9 kΩ values, so an individual ΔR/R value is quantised in steps of about 0.1%; the increments of +0.09 and +0.15 pp correspond to one to three such steps at the resistor level and are therefore reported to two decimals only.

**Figure 4:**
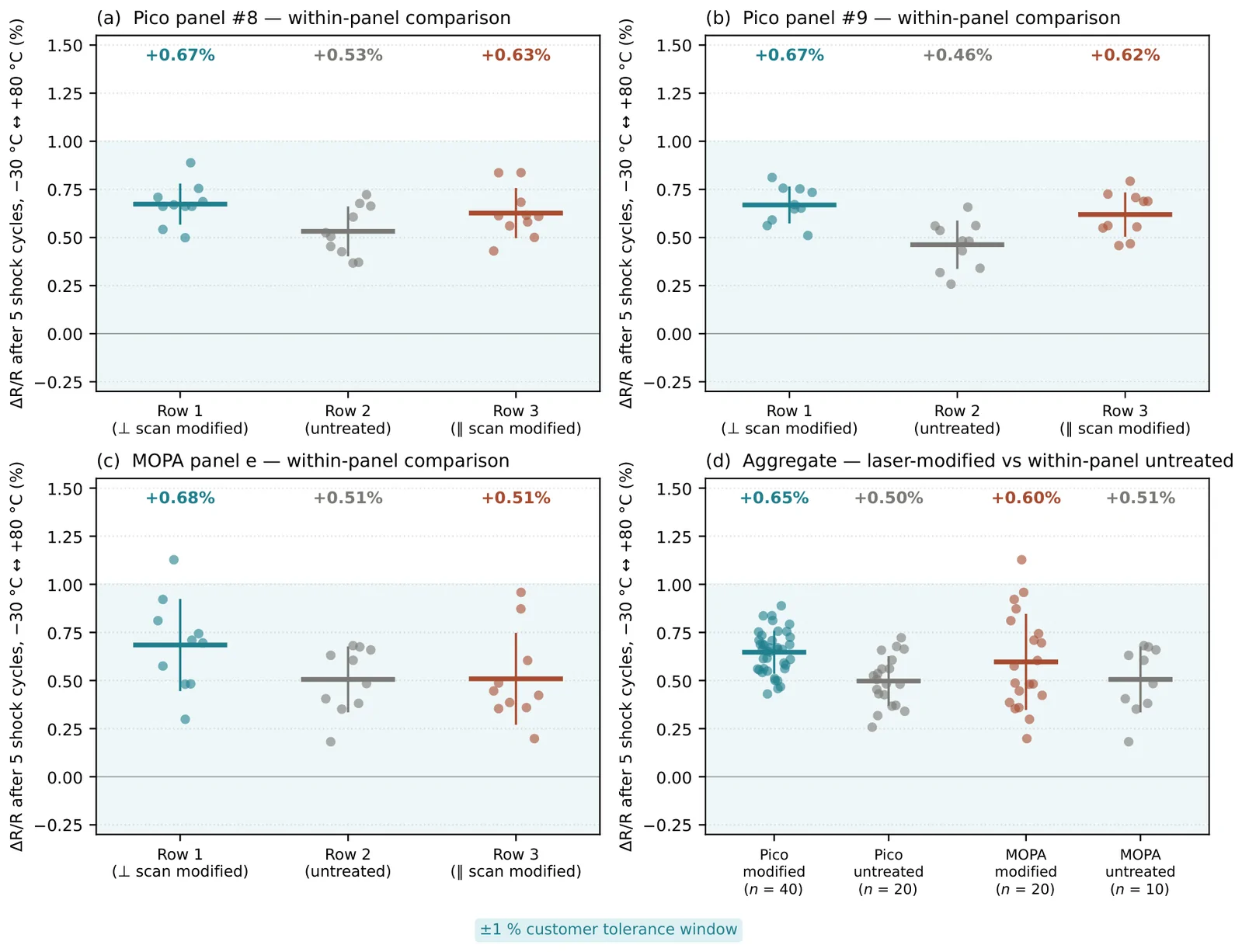
Thermal-shock ΔR/R after five cycles between −30 °C and +80 °C for each resistor row of each panel: laser-modified Row 1 (⊥, scan perpendicular to the current path, teal), co-fired within-panel untreated Row 2 (grey), and laser-modified Row 3 (‖, scan parallel to the current path, terra). (**a**) Pico panel #8, (**b**) Pico panel #9, (**c**) MOPA panel e, (**d**) aggregate across panels. Horizontal bar = mean; vertical whisker = ±1SD. Shaded band shows the ±1% customer tolerance window. Fifty-nine of sixty laser-modified resistors (40/40 Pico and 19/20 MOPA) and all thirty within-panel untreated resistors remain inside ±1%; the single MOPA excursion was +1.13%. The MOPA panel (**c**) is preliminary feasibility evidence from one panel. The laser-associated increment vs. the within-panel untreated row is +0.15 pp (Pico) and +0.09 pp (MOPA); the baseline drift of the untreated Row 2 itself is +0.50%, a property of the 8P7R lot used as experimental vehicle rather than of the laser modification.

**Reading of the result.** On the same 8P7R lot and the same shock protocol, the within-panel untreated resistors themselves drift by mean +0.50% after five cycles between −30 °C and +80 °C. This is a property of this lot of commercial 8P7R arrays—an experimental vehicle chosen for its regular geometry and same-panel controls, not an IEC 60115-1-qualified high-reliability part—and it is not attributable to laser modification. The laser-modified rows sit at +0.60–0.65% under the identical shock exposure, i.e., within +0.09 pp (MOPA) to +0.15 pp (Pico) of the within-panel untreated control. Fifty-nine of the sixty laser-modified resistors and all thirty untreated control resistors remain inside ±1% (Pico: 40/40 modified and 20/20 untreated; MOPA: 19/20 modified and 10/10 untreated; the single MOPA excursion is at +1.13%, i.e., 0.13 pp above the ±1% line, on a resistor whose within-panel untreated control on the same panel drifted +0.51%). Figure 4 shows the distribution.

**Equivalence test on the thermal-shock increment.** Failing to detect a difference does not by itself demonstrate the absence of a penalty, so a TOST procedure was applied to the modified-minus-untreated increment with an equivalence margin of ±0.5 pp. At the resistor level (Welch standard error, descriptive of the tested panels), the 90% confidence interval of the increment is [+0.09,+0.21] pp for Pico (n=40 vs. 20; TOST p<0.001) and [−0.04,+0.22] pp for MOPA (n=20 vs. 10; TOST p<0.001). Because resistors are nested in panels, the Pico increment was also evaluated with the panel as the unit: the within-panel increments are +0.12 pp (panel #8) and +0.18 pp (panel #9), giving a mean of +0.15 pp with a 90% confidence interval of [−0.05,+0.35] pp (1 degree of freedom), still inside the ±0.5 pp margin (TOST p=0.03). A panel-level analysis is not possible for MOPA, which has only one panel. These results are consistent with no practically relevant thermal-shock penalty under the tested conditions, but the margin was defined by engineering convention rather than pre-registered, and the exposure (five cycles, −30/+80 °C) is short and mild.

**Conclusion of Section 3.3.** On the tested 8P7R panels and under the five-cycle −30/+80 °C exposure, the laser-modified rows drifted on average 0.15 pp (Pico) and 0.09 pp (MOPA) more than the within-panel untreated rows. The Pico observations span two panels; the MOPA observations come from one panel and are *preliminary feasibility evidence* only, not a population-level route comparison. Transfer to embedded resistors in a high-value multilayer HIC substrate, and to more severe thermal-cycling conditions, is discussed in Section 4.6.

### 3.4. TCR Evolution Across Five Processing Stages

**Figure 5:**
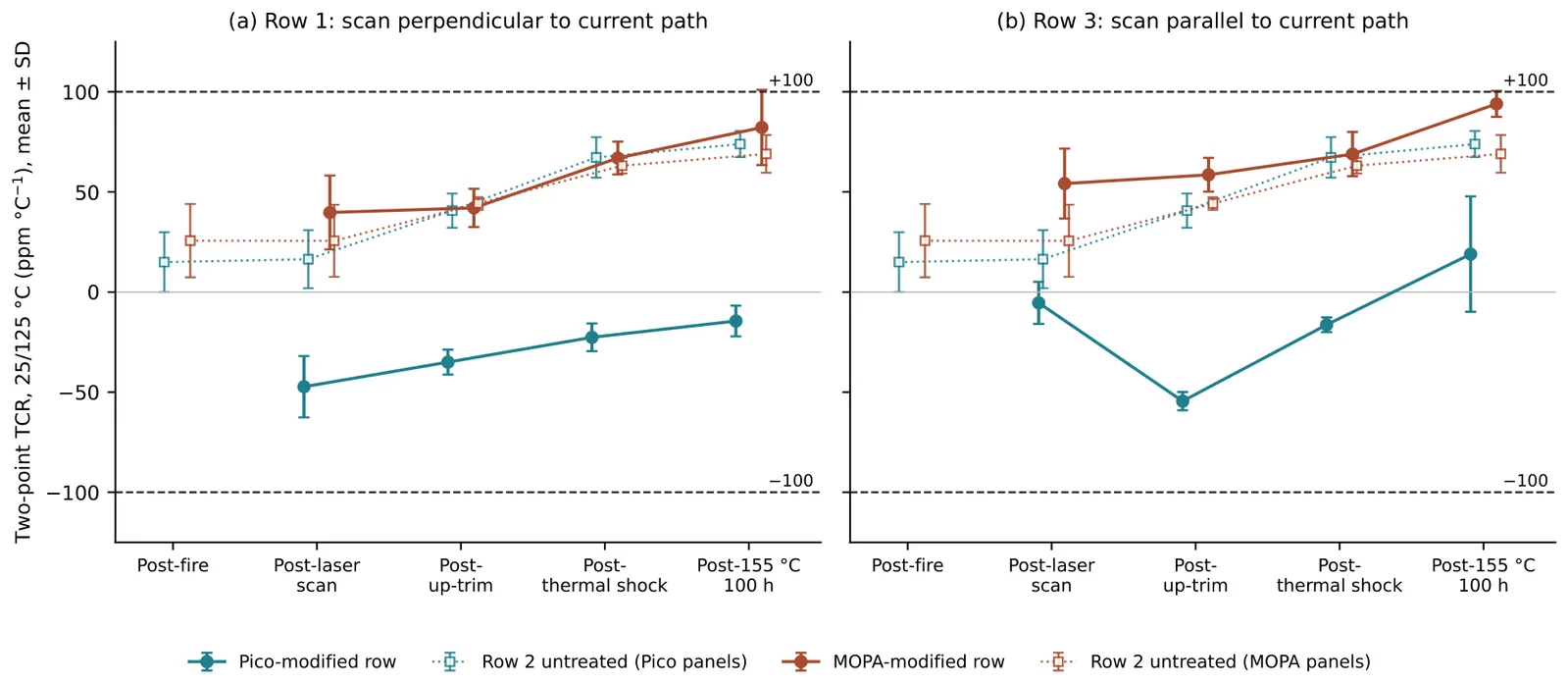
Evolution of the temperature coefficient of resistance across the five processing stages—post-fire, post-laser modification, post-up-trim, post-thermal-shock, and post-155 °C × 100 h high-temperature storage—for (**a**) Row 1 and (**b**) Row 3 (scan lines perpendicular and parallel to the current path, respectively). Points are stage means ±1 SD; successive stages were measured on different resistor sets (see text), so the lines connect stage means rather than same-resistor trajectories. Dashed lines: ±100 ppm °C^−1^. TCR is the two-point 25/125 °C value measured once per resistor and stage (no repeat measurements). The Row 2 within-panel untreated rows of the Pico and MOPA panels use dotted lines with open square markers, whereas both laser trajectories use solid lines with circular markers, preserving identification in monochrome printing.

The temperature coefficient of resistance of RuO_2_-glass thick-film resistors reflects the balance between metallic-like conduction in the RuO_2_ percolation network and the glassy matrix that surrounds it, and is well described by the percolation and mixed-conductivity frameworks of Kusy [5], Crosbie [6], and Simonnet [31]; it is the second reliability metric that determines whether a resistor stays within its performance specification over its service life. Figure 5 tracks the mean TCR through five sequential processing stages—post-fire (as-fired), post-laser modification, post-up-trim, post-thermal-shock (five cycles −30 to +80 °C), and post-155 °C × 100 h high-temperature storage—for both the perpendicular-scan row (Row 1) and the parallel-scan row (Row 3), together with the untreated Row 2 of the Pico and of the MOPA panels.

The stages were measured on different resistor sets: at post-fire, four resistors per row on each route panel (n=44 per Pico row, n=16 per MOPA row); at post-laser, fourteen resistors per row on each route panel (n=154 per Pico row, n=56 per MOPA row); after up-trim, Pico panel #2 and MOPA panel *e* (n=10 per row); after thermal shock, Pico panels #8 and #9 (n=20) and MOPA panel *e* (n=10); after 155 °C storage, Pico panel #5 and MOPA panel *f* (n=10). The curves in Figure 5 therefore connect stage means and are not same-resistor trajectories. Four observations characterise the data:

(i) The untreated Row 2, which was also up-trimmed (+50%) and exposed to the same thermal steps, rises from 14.9 to 16.4, 40.7, 67.2, and 73.9 ppm °C^−1^ on the Pico panels and from 25.6 to 25.6, 44.2, 63.1, and 69.0 ppm °C^−1^ on the MOPA panels. This rise occurs without laser modification and forms the within-panel baseline for each route.

(ii) In Row 1, the Pico TCR moves from −47.3 (post-laser) to −35.0, −22.6, and −14.5 ppm °C^−1^; its magnitude therefore decreases by about 70%. Because the untreated baseline rises faster, however, the offset from the Pico-panel Row 2 widens from −63.7 ppm °C^−1^ after laser modification to −88.4 ppm °C^−1^ after storage. In Row 3 the Pico TCR moves from −5.4 to −54.5, −16.3, and +19.0 ppm °C^−1^, ending −55.0 ppm °C^−1^ below its Row 2 baseline.

(iii) In Row 1, the MOPA TCR moves from +39.7 to +42.0, +66.9, and +82.2 ppm °C^−1^, and in Row 3 from +54.2 to +58.6, +68.9, and +94.0 ppm °C^−1^. Relative to the MOPA-panel Row 2, the offset is +14.1 (Row 1) and +28.6 ppm °C^−1^ (Row 3) after laser modification and +13.2 and +25.0 ppm °C^−1^ after storage, i.e., the MOPA rows rise together with the baseline.

(iv) At the resistor level, every measured Pico value remained within ±100 ppm °C^−1^ at all stages (after storage: Row 1 minimum −34.2, Row 3 maximum +56.5 ppm °C^−1^). The MOPA values remained within ±100 ppm °C^−1^ up to and including thermal shock (maximum +80.0 ppm °C^−1^); after 155 °C storage, Row 1 had 10/10 within the limit (maximum +96.8 ppm °C^−1^), whereas Row 3 had 8/10 within the limit, with two resistors at +100.9 and +107.0 ppm °C^−1^.

In practical terms, the stage-mean TCR of both routes stayed inside ±100 ppm °C^−1^ through the sequence, and the Pico route kept every measured resistor inside that limit. The MOPA route approached the upper limit after high-temperature storage, where 2 of 10 Row 3 resistors exceeded it; a MOPA route would therefore need an explicit post-ageing TCR check against the customer specification. Two measurement limits apply: First, the resistances used for TCR after up-trim, thermal shock, and storage were recorded with 1 Ω resolution (post-fire and post-laser: 0.01 Ω), so individual TCR values at those later stages are quantised in steps of roughly 5–10 ppm °C^−1^ depending on resistance. Second, each value is a two-point chord TCR measured once per resistor and stage, without repeat measurements or intermediate temperatures. Stage-to-stage changes of the order of the reported standard deviations are therefore within the combined resistor-to-resistor and measurement scatter and are not interpreted mechanistically.

### 3.5. Direct Material Verification (SEM and EDS)

**Figure 6:**
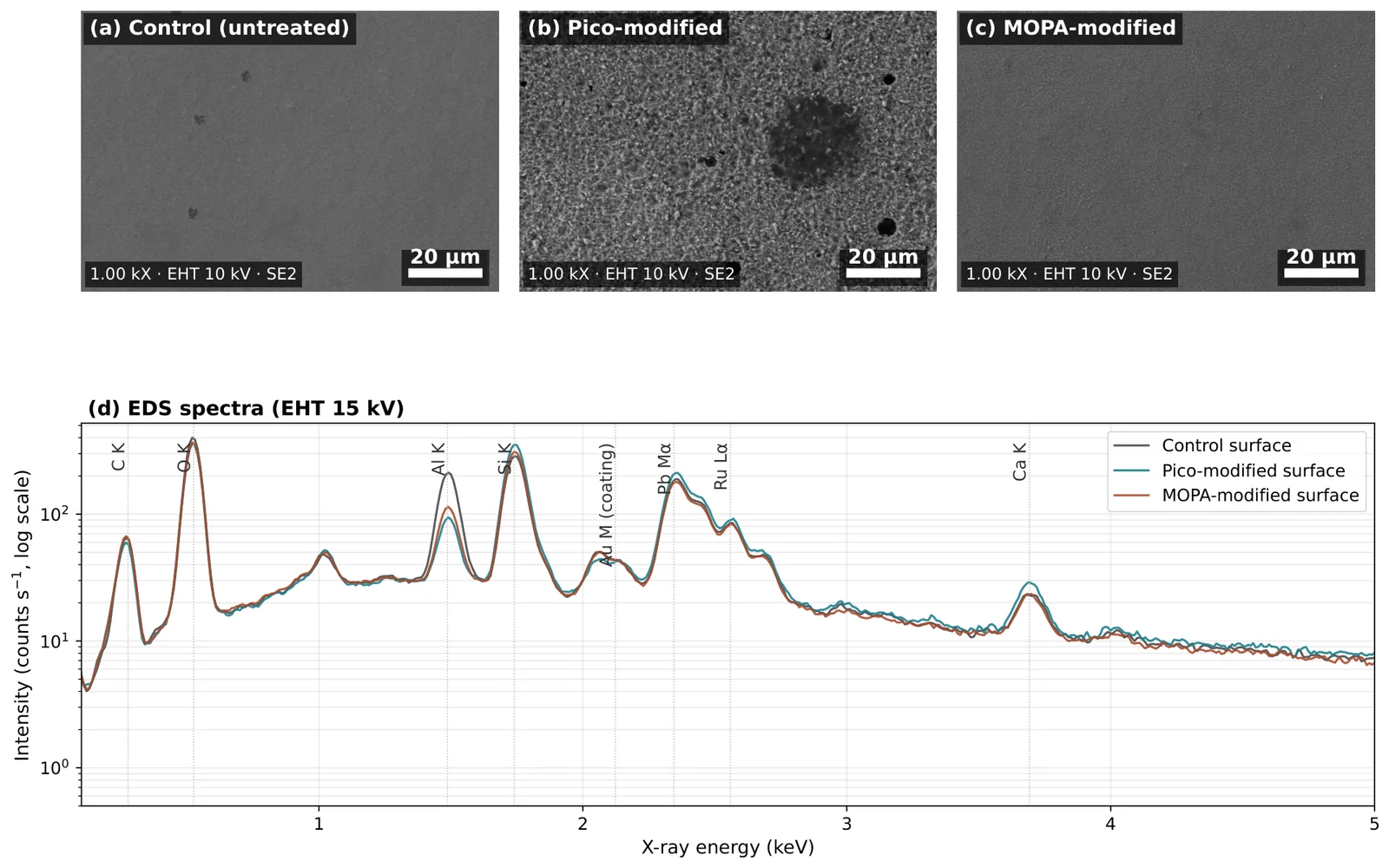
Direct-material verification of the two laser routes. (**a**–**c**) Top-view SEM at 1 kX (EHT=10 kV, SE2 detector) of the untreated control, Pico-modified, and MOPA-modified surfaces (one representative field per condition); both modified surfaces retain the granular network of the underlying resistor film. (**d**) Corresponding EDS spectra; the four principal peaks O K, Si K, Pb Mα, and Ru Lα of the RuO_2_–PbO–SiO_2_ resistor system remain detectable on all three surfaces; EDS does not establish chemical-phase preservation.

To check that the electrical results of Section 3.1, Section 3.2, Section 3.3 and Section 3.4 were obtained on an intact resistor stack, top-view SEM imaging at 1 kX (EHT = 10 kV, SE2 detector) and EDS were performed on three surface conditions: untreated control, Pico-modified, and MOPA-modified. The specimens were taken from the panels listed in row 13 of Table 1; Figure 6 shows one representative top-view field per condition, taken on the resistor surface (for the modified conditions, within the scanned area and away from any up-trim kerf). Only top-view imaging was performed; no cross-sections were prepared, and no area-weighted statistics were extracted from the images.

**Direct observations**.

Figure 6a–c shows the surface morphology of the three conditions. The untreated control (Figure 6a) exhibits a compact, fine-textured surface with only minor isolated porosity, characteristic of a correctly co-fired RuO_2_–PbO–SiO_2_ film. The Pico-modified surface (Figure 6b) exhibits a strongly reworked morphology with sub-micron re-solidification texture and a locally reworked crater feature; the MOPA-modified surface (Figure 6c) is smoother than the Pico surface but coarser than the control. Both modified surfaces retain the granular network of the underlying resistor film and do not exhibit macroscopic delamination or debonding.

The corresponding EDS spectra (Figure 6d) confirm retention of the principal elemental constituents after both modification routes. The four principal peaks that define the resistor system—O K (0.53 keV), Si K (1.74 keV), Pb Mα (2.35 keV), and Ru Lα (2.56 keV)—are present on all three surfaces with intensities that are within a factor of two of the control. The elemental signatures associated with the Ru-containing conductive component and the Pb–Si–O glass matrix therefore remain detectable after both laser modifications. EDS alone does not establish phase identity; confirmation of RuO_2_ phase preservation would require a phase-sensitive method such as Raman spectroscopy or XRD. An Al K peak (1.49 keV) is also present and is attenuated on the two modified surfaces. At 15 kV, the X-ray generation depth in such an oxide film is of the order of 1–2 μm, so a contribution from the alumina substrate is unlikely unless the film is locally very thin; the signal more plausibly originates from Al-containing glass or filler constituents of the paste. Its origin was not determined here. A Ca K peak (3.69 keV) is also visible in all three spectra; it is tentatively attributed to Ca-containing glass constituents of the paste, but its origin was likewise not determined.

**Literature-based interpretation (not measured here).** The attenuation of the Al K signal is compatible with a re-solidified overlayer on the modified surfaces, but its thickness was not measured.

These observations permit only a qualitative interpretation of the resistance decrease. The re-solidification texture seen by SEM demonstrates that both lasers thermally reworked the resistor surface rather than merely producing a conventional open kerf, while the continued Ru, O, Pb, and Si signatures show that the reworked region still contains constituents of both the conductive component and glass matrix. This combination is consistent with the mechanism proposed by Gofuku et al.—laser-driven modification of the glass and incorporation of Ru-related species that increases electronic conduction [25]. However, the present top-view SEM/EDS data do not measure glass conductivity, Ru spatial redistribution, crystalline phase, or Ru valence; therefore, they support that mechanism only as a plausible interpretation, not as proof. Cross-sectional elemental mapping together with XRD, Raman, or XPS would be required for direct mechanistic confirmation.

Future mechanistic work should add cross-sectional SEM/EDS mapping of the modified-layer thickness and a phase- and valence-sensitive analysis of the Ru-containing region.

## 4. Discussion

The four production-related metrics reported in Section 3 support a single conclusion of practical importance to thick-film resistor production: an over-target RuO_2_ thick-film resistor, which cannot be corrected by conventional one-way up-trimming, can first be shifted below target by surface modification and then returned accurately to target by ordinary closed-loop trimming. The data demonstrate this dual-step recovery function on the 8P7R experimental vehicle. They support follow-on qualification rather than establishing IEC conformity or direct transferability to embedded HIC resistors. The choice between the two therefore does not rest on any single go/no-go reliability threshold but on the combined production profile summarised in Table 6 and discussed in detail below.

### 4.1. Recovery Capability of the Two Routes

The immediate benefit of using either laser modification (Section 3.1) is that a resistor whose fired value is above the customer tolerance window can be brought below target and then trimmed back into the window without scrapping the substrate on which it is fabricated. In the target application—a multilayer HIC—this benefit is amplified by the high per-unit cost of the assembly: given that the post-fire resistance distribution of a thick-film printing can produce out-of-tolerance units at the ±1% customer window, each recovered HIC substrate avoids scrapping an assembly that already carries its co-fired ceramic, thin-film, and paste cost.

The two recipes differ in how much of this recovery they cover. The Pico recipe lowered every tested resistor to Rf/Ri ≤0.681 and therefore covered a +15% over-target case for all 3192 resistors. The MOPA recipe lowered 77.6% of resistors to Rf/Ri ≤0.870; for the remaining 22.4%, a single pass under the tested recipe would cover only a smaller over-target deviation (down to about +2.6% for the highest observed ratio). In both cases the closed-loop up-trim step, not the modification step, sets the final ±1% tolerance (Section 3.2).

### 4.2. Why Similar Up-Trim Precision Was Observed

On the tested panels, the Pico–MOPA differences in up-trim result fell within the $\pm 5\times 10^{-3}$ trim-ratio margin (Section 3.2), although the two modification recipes differ by about three orders of magnitude in pulse duration (10–12 ps versus ≈6 ns). This is consistent with the architecture of the process: the modification lasers never cut the up-trim kerf. Every kerf on every panel was cut by the same shared trimmer with the same closed-loop resistance feedback, which stops the cut when the measured resistance reaches the target. The final trimmed value is therefore governed mainly by the trimmer and its feedback loop, and the observed agreement suggests that neither modification recipe left the film in a state that disturbs this closed-loop trimming. For the manufacturer, this means that either modification route is compatible with an existing up-trim line, provided that the kerf geometries that did not hold the ±1% window in this study (the U-cut, and the L-cuts beyond +25%) are avoided.

### 4.3. Route-Specific Differences

The two laser sources are not identical in their post-modification signature, however. Two secondary properties differ measurably and are relevant to production planning:

**Thermal-shock behaviour of the modified stack, referred to the within-panel untreated control (Section 3.3, Figure 4).** On per-resistor paired ΔR/R data (*n* = 90 resistors, three rows on two Pico panels and one MOPA panel), the laser-modified rows drift by +0.60–0.65% after five shock cycles (−30/+80 °C), and the co-fired untreated row (Row 2) of the same panel drifts by +0.50%. The increment associated with laser modification is therefore +0.15 pp for the Pico route and +0.09 pp for the MOPA route. These are descriptive contrasts for the tested panels, not population-level estimates. Fifty-nine of the sixty laser-modified resistors and all thirty untreated controls stay inside ±1% (Pico: 40/40 modified + 20/20 untreated; MOPA: 19/20 modified + 10/10 untreated). This within-panel reading is the natural reliability comparator for a method-validation study: it separates the observed modified-minus-control contrast from the baseline thermal-shock behaviour of the 8P7R lot itself. The difference between the post-trim and post-thermal-shock TCR stage means of the modified rows stays under +40 ppm °C^−1^ on both routes (for Pico, the two stages were measured on different panels, so this is a difference in stage means rather than a same-resistor change), and all modified-row stage means remain inside ±100 ppm °C^−1^; at the resistor level, 2 of 10 MOPA Row 3 resistors exceeded +100 ppm °C^−1^ after 155 °C storage (Section 3.4).

**Temperature-coefficient trajectory during ageing (Section 3.4, Figure 5).** Pico-modified resistors carry a negative TCR (−47 ppm °C^−1^ after laser, Row 1) that moves towards zero during ageing (−14 ppm °C^−1^ after 155 °C × 100 h storage), while MOPA-modified resistors carry a positive TCR (+40 ppm °C^−1^ after laser, Row 1) that grows with high-temperature storage (+82 ppm °C^−1^ after 155 °C × 100 h). Both Row 1 endpoints remain inside the ±100 ppm °C^−1^ limit, whereas the MOPA Row 3 endpoint reached it at the resistor level, and the two routes call for different checks:

- For Pico-modified resistors, the TCR check is most critical immediately after modification, since the negative Row 1 stage mean moved towards zero at the later stages.
- For MOPA-modified resistors, the TCR check is most critical after high-temperature storage, since the stage mean rose at each step after up-trim.

The within-panel untreated Row 2 rises by about +59 ppm °C^−1^ (Pico panels) and +43 ppm °C^−1^ (MOPA panels) across the same stages, and this drift—a property of the resistor paste itself and independent of the laser modification—is the baseline against which any laser row must be compared. The practical consequence is that the manufacturer should specify the TCR-test protocol (post-laser vs. post-ageing) explicitly in the customer part-number for either laser route.

### 4.4. Qualitative Interpretation of the Resistance-Lowering Step

Section 3.5 (Figure 6) shows that the electrically measured resistance decrease accompanies a reworked and re-solidified surface morphology without macroscopic delamination. The O, Si, Pb, and Ru EDS signatures remain detectable on both modified surfaces. Taken together, these observations are qualitatively compatible with the Ru-containing glass-modification model reported by Gofuku et al. [25], and they argue against gross film loss or debonding as the useful operating state. They do not, however, identify the microscopic conduction mechanism. In particular, EDS cannot establish Ru valence or distinguish RuO_2_ from Ru incorporated into a modified glass phase. The present paper therefore treats the mechanism as a literature-supported explanation and confines its own claim to the measured two-step recovery function and associated reliability evidence.

### 4.5. Production-Planning Summary

Table 6 consolidates the quantitative results of Section 3.1, Section 3.2, Section 3.3, Section 3.4 and Section 3.5 into a single production-planning view. It is intended as an at-a-glance reference for HIC process engineers deciding which laser route to deploy at the follow-on substrate-qualification step, and it aligns the recovery, trim-precision, thermal-shock, TCR, and material-integrity criteria on a per-route basis so that the trade-offs are directly comparable.

### 4.6. Method-Validation Reading and Follow-On Qualification for HIC Transfer

**Scope of this study.** The industry motivation of this work is the recovery of post-fire out-of-tolerance embedded resistors on high-value multilayer HIC substrates by laser modification followed by conventional up-trim. Because a mis-processed HIC substrate carries the full accumulated cost of its upstream co-fired-ceramic and thin-film processing, the laser-modification methodology has to be validated on a controlled experimental vehicle before it is committed to production HIC panels. The 8P7R SIP-8 resistor array was chosen for that role because it is fabricated by the same screen-print, co-fire, and laser-trim process used inside HICs, provides three co-fired resistor rows on a single panel (permitting a within-panel untreated control), and is independent of downstream HIC assembly cost. The results of Section 3.1, Section 3.2, Section 3.3, Section 3.4 and Section 3.5 answer the four method-validation questions of the Introduction on this vehicle:

1. Both laser routes lowered over-target resistors below target; the Pico recipe did so for a +15% case in every tested resistor and the MOPA recipe in 77.6% of resistors, after which the shared up-trim returned them to the ±1% window, fully with the scan-cut and with at most 4 of 34 resistors per row outside for the I-, D-, and M-cuts (Section 3.1 and Section 3.2);
2. **On the tested panels, the two routes gave up-trim results within a $\pm 5\times 10^{-3}$ engineering margin across seven kerf geometries on the shared trimmer** (descriptive TOST; Section 3.2);
3. **The Pico route remained within 0.15 pp of its same-panel untreated control in thermal shock; the one-panel MOPA observation was similarly close but is preliminary feasibility evidence**, and 59 of 60 laser-modified resistors stayed inside ±1% across the tested panels and the five-cycle −30/+80 °C exposure (Section 3.3);
4. The stage-mean TCR of both routes stayed inside ±100 ppm °C^−1^ through the five-stage sequence, with different trajectories; at the resistor level, 2 of 10 MOPA Row 3 resistors exceeded +100 ppm °C^−1^ after 155 °C storage (Section 3.4).

The direct-material observations of Section 3.5 (SEM + EDS) show no macroscopic delamination and retain the principal elemental signatures for both routes. The two tested scan orientations (Row 1 perpendicular, Row 3 parallel) produced similar thermal-shock responses on the tested panels; this is presented as feasibility evidence rather than a formal orientation-equivalence result.

**What the manuscript does*****not*** **claim.** This study is a feasibility validation of the laser-modification methodology on an experimental vehicle; it is not a screening study of an IEC 60115-1-qualified high-reliability part. The 8P7R lot used here shows a baseline thermal-shock drift of mean +0.50% on its untreated resistors (Section 3.3, Row 2), which is a property of the commercial 8P7R part chosen as vehicle, not of the laser modification. No claim of IEC 60115-1 qualification is made for the 8P7R lot itself, and none is required by the method-validation objective. Similarly, this study does not claim that one kerf geometry or scan orientation is superior to another; these variants are used to show that the method is verifiable across them, not to rank them.

**Structural differences between the 8P7R vehicle and embedded HIC resistors.** The 8P7R results cannot be transferred to embedded HIC resistors without re-verification, because the two constructions differ in at least the following respects (Table 7). None of these differences was measured in the present study; the table states what each difference could change and what must be re-verified.

**Follow-on qualification for HIC transfer.** The natural follow-on work is a targeted re-validation of the modification recipe on dedicated multilayer HIC substrates, where the customer specification may require a tighter (e.g., ±0.5%) thermal-shock window on the finished product. Prior work on embedded thick-film resistors in low-temperature co-fired ceramic (LTCC) and multilayer hybrid substrates [1,2,3,4,7] shows that the embedded form factor changes the trim-feedback path and the thermal boundary condition relative to a surface-printed resistor. The follow-on study should therefore re-run the four production-related metrics on the substrate class of interest, with multiple independent panels per route, a longer and more severe thermal-cycling profile, repeated TCR measurements with intermediate temperatures, and a sampling plan appropriate to the customer specification.

## 5. Conclusions

We have validated the feasibility of a laser-modification-plus-trim recovery methodology for post-fire out-of-tolerance RuO_2_-based thick-film resistors, on a controlled 8P7R SIP-8 experimental vehicle, using two complete surface-modification recipes (picosecond and nanosecond MOPA) with two scan orientations per panel (perpendicular Row 1 and parallel Row 3), a single shared trimmer for all up-trim kerfs, and a co-fired within-panel untreated row (Row 2) that acts as the reliability comparator on the same substrate, package, paste, and shock exposure. On raw data from 15 unique 8P7R panels (11 Pico + 4 MOPA), 6540 unique physical resistors had valid paired pre- and post-process measurements; 75 records with printing defects were excluded before Rf/Ri calculation. The main observations are as follows:

1. **Both dual-step routes lowered over-target resistors below target, after which closed-loop up-trimming restored them to target.** Pico surface modification brought the modified resistor to 61% of the as-fired value (Rf/Ri = 0.607 ± 0.015, *n* = 3192; every resistor ≤0.870, i.e., sufficient for a +15% over-target case); MOPA surface modification to 83% (Rf/Ri = 0.831 ± 0.052, *n* = 1175), with 77.6% of resistors at ≤0.870, so the tested MOPA recipe covers a +15% case for about three quarters of resistors. No overshoot (Rf > Ri) occurred. The scan-cut kept every resistor within ±1%, the I-, D-, and M-cuts had at most 4 of 34 resistors per row outside it, and the U-cut did not hold it and is not recommended for production. Reversing the normal one-way trimming limitation in this way is the central manufacturing contribution of the work.
2. **On the tested panels, the Pico–MOPA up-trim differences fell within the engineering margin across seven kerf geometries on the shared trimmer** (paired TOST on the 15 matched cells in which both route means lie within ±1% of target, margin $\pm 5\times 10^{-3}$ in trim ratio, mean difference $+1.6\times 10^{-3}$, p<0.001; Pico/MOPA SD ratio 0.99); the margin was exceeded when the kerf and increment combinations that did not reach the target were included. Because the cells come from a limited number of panel rows and are not independent panel replicates, this is descriptive engineering evidence, not population-level proof of route equivalence.
3. **The Pico route showed thermal-shock response close to its same-panel untreated control; the one-panel MOPA result is preliminary feasibility evidence.** On per-resistor paired ΔR/R measurements the laser-modified rows drift by +0.60–0.65% after five cycles between −30 °C and +80 °C, and the co-fired untreated Row 2 of the same panel drifts by +0.50%. The laser-induced descriptive modified-minus-control contrast is +0.15 pp for the Pico route (within the ±0.5 pp engineering equivalence margin at both the descriptive resistor level and the panel level) and +0.09 pp for the MOPA route. Fifty-nine of the sixty laser-modified resistors and all thirty within-panel untreated resistors remain inside ±1% across the shock protocol on both routes. This is the natural feasibility observation for the tested panels: the laser modified rows remained close to the untreated row from the same 8P7R lot under the tested conditions. Both tested scan orientations produced similar feasibility outcomes, but the experiment was not designed as a formal orientation-equivalence test. In particular, the MOPA thermal-shock dataset comprises one make-up panel and must not be interpreted as population-level reliability evidence or as a comparative ranking against Pico.
4. **The stage-mean two-point (25/125 °C) TCR of both routes stayed inside ±100 ppm °C^−1^** through the five-stage sequence (post-fire → post-laser → post-trim → post-thermal-shock → post-155 °C × 100 h storage). The Pico Row 1 value was negative and moved toward zero, while its offset from the rising untreated Row 2 widened; the MOPA values were positive and rose with the baseline after up-trim. At the resistor level, all Pico values remained within ±100 ppm °C^−1^, whereas 2 of 10 MOPA Row 3 resistors exceeded +100 ppm °C^−1^ (+100.9 and +107.0) after 155 °C storage. The stage values come from different resistor sets, and the TCR-test stage should be specified in the customer part number for either route.
5. **Both routes retain the O–Si–Pb–Ru elemental signatures on top-view EDS** and produce no macroscopic delamination on top-view SEM. The reworked and re-solidified morphology is qualitatively consistent with the published Ru-containing glass-modification mechanism, but phase, valence, and carrier formation were not measured and are not claimed as proven here.

**Scope note.** The 8P7R experimental vehicle used in this study is a commercial resistor array chosen for its regular geometry and its co-fired within-panel controls, not an IEC 60115-1-qualified high-reliability part; the baseline thermal-shock drift of mean +0.50% on its untreated Row 2 is a property of the vehicle, not of the laser modification. Correspondingly, this paper does not claim IEC 60115-1 qualification for the 8P7R lot itself, and does not rank kerf geometries against one another. What it does report is that the laser-modification methodology produced thermal-shock responses close to the same-panel untreated controls on the tested 8P7R panels.

**Limitations.** (i) The panel, not the resistor, is the independent replicate; with two Pico panels and one MOPA panel in thermal shock and one panel row per up-trim cell, the results are descriptive of the tested panels, and the MOPA thermal-shock result is preliminary feasibility evidence. (ii) The two modification recipes differ in pulse duration, power, and lens simultaneously, so the recovery difference is a recipe-level result. (iii) The thermal-shock exposure was five cycles between −30 and +80 °C, which is a short screening condition, not a qualification test. (iv) TCR was measured by a two-point method, once per resistor and stage, without repeat measurements or intermediate temperatures; measurement repeatability and R(T) curvature were therefore not quantified. (v) The mechanism of the resistance decrease was not isolated; SEM/EDS was top-view only, on one representative field per condition. (vi) Resistances used for TCR after up-trim, thermal shock, and storage were recorded with 1 Ω resolution (post-fire and post-laser: 0.01 Ω), so individual TCR values at those stages are quantised in steps of roughly 5–10 ppm °C^−1^, and the TCR stages were measured on different resistor sets rather than on the same resistors. (vii) The initial thermal-shock records of panels 4 and *h* were not retained or are incomplete, so these panels were not used in the reported thermal-shock analysis, which relies on panels #8, #9, and *e*; both panels are included in the Rf/Ri and post-laser TCR data. (viii) An earlier hand-compiled summary of the up-trim data differed from the per-panel records in some cells; Table 3 and all up-trim statistics were therefore regenerated directly from the per-panel records, with the panel *e* Row 1 U-cut record excluded as a recording error. (ix) Post-shock resistances were also recorded with 1 Ω resolution, so individual thermal-shock ΔR/R values are quantised in steps of about 0.1%, comparable to the reported increments.

**Transfer to high-value multilayer HICs.** With the feasibility of the method shown on 8P7R, the follow-on qualification step is a targeted re-run of the four production-related metrics on dedicated multilayer HIC substrates, where the embedded resistors are screened parts and the customer specification may require a ±0.5% thermal-shock window on the finished product. The present results support proceeding to that substrate-level evaluation; they do not replace it.

## Figures and Tables

**Table 1:** Panel allocation and experimental purpose.

#	Panel IDs	Count	Role	Notes
1	2–9, b–d	11 panels	Pico surface modification (area scan of Rows 1 and 3)	—
2	e–h	4 panels	MOPA surface modification (area scan of Rows 1 and 3)	—
3	j, k	2 panels	Untreated control	Provides ground-truth as-fired baseline
4	2–9, b–d	11 panels	Pico 125 °C TCR characterisation	—
5	e–h	4 panels	MOPA 125 °C TCR characterisation	—
6	2–9, b–d	11 panels	Pico up-trim at +25%, +50%, +75% (shared trimmer)	Up-trim analysis (Section 3.2) uses panels #2 and #5
7	e–h	4 panels	MOPA up-trim at +25%, +50%, +75% (shared trimmer)	Up-trim analysis (Section 3.2) uses panels *e* and *f*
8	j, k	2 panels	Control up-trim (verification)	Not used in the reported analyses
9	4, g, h	3 panels	Thermal-shock initial	Panel *g* Row 1 showed a mis-registered kerf and was excluded; the initial-run records of panels 4 and *h* were not retained or are incomplete and were not used; the reported analysis relies on rows 10–11
10	8, 9	2 panels	Thermal-shock final (Pico)	Added 10 November 2025 to complete Pico paired-ΔR/R dataset
11	*e*	1 panel	Thermal-shock (MOPA)	MOPA make-up run on panel *e*; per-resistor ΔR/R paired position-by-position across all three rows
12	5, j, f	3 panels	155 °C × 100 h high-temperature storage	Pico (5), control (j), MOPA (f)
13	2, 6, e, 7, h	5 panels	SEM/EDS material characterisation	See Section 3.5 for site allocation

**Table 2:** Panel-level Rf/Ri after the resistance-lowering step (Rows 1 and 3 of each panel). Last column: resistors with Rf/Ri ≤0.870, i.e., able to bring a resistor 15% above target below target.

Route	Panel	*n*	Mean	SD	Range	*n* (Rf/Ri ≤0.870)
Pico	#2	294	0.616	0.013	0.588–0.647	294 (100.0%)
Pico	#3	294	0.605	0.013	0.573–0.640	294 (100.0%)
Pico	#4	294	0.605	0.013	0.580–0.642	294 (100.0%)
Pico	#5	287	0.602	0.014	0.560–0.655	287 (100.0%)
Pico	#6	294	0.603	0.021	0.551–0.673	294 (100.0%)
Pico	#7	280	0.610	0.011	0.584–0.634	280 (100.0%)
Pico	#8	273	0.611	0.021	0.566–0.681	273 (100.0%)
Pico	#9	294	0.605	0.009	0.581–0.632	294 (100.0%)
Pico	*b*	294	0.602	0.012	0.576–0.632	294 (100.0%)
Pico	*c*	294	0.615	0.010	0.591–0.643	294 (100.0%)
Pico	*d*	294	0.604	0.014	0.566–0.635	294 (100.0%)
MOPA	*e*	294	0.843	0.051	0.770–0.971	214 (72.8%)
MOPA	*f*	294	0.834	0.049	0.751–0.968	225 (76.5%)
MOPA	*g*	294	0.843	0.050	0.769–0.975	215 (73.1%)
MOPA	*h*	293	0.804	0.047	0.729–0.921	258 (88.1%)
Pico	all	3192	0.607	0.015	0.551–0.681	3192 (100.0%)
MOPA	all	1175	0.831	0.052	0.729–0.975	912 (77.6%)

**Table 3:** Up-trim results per laser route × kerf geometry × row, regenerated from the per-panel records (Pico: panels #2 and #5; MOPA: panels *e* and *f*; interior positions 3–19 of each row). Entries: mean ratio (SD $\times \;10^{3}$), bias, and number of resistors outside ±1% of target/*n*. Ratio = post-trim/pre-trim resistance; bias = (mean ratio − target)/target. Row 1: modified, scan perpendicular to the current path, target 1.25; Row 2: untreated, target 1.50; Row 3: modified, scan parallel to the current path, target 1.75. ^a^ Panel *f* only; the panel *e* record contains ratios below 1 and was excluded as a recording error.

Kerf	Row	Target	Pico: Mean (SD $\times \;10^{3}$), Bias, out/*n*	MOPA: Mean (SD $\times \;10^{3}$), Bias, out/*n*
I-cut	1	1.25	1.2541 (2.56), +0.32%, 0/34	1.2532 (2.73), +0.26%, 0/34
	2	1.50	1.5050 (10.67), +0.33%, 1/34	1.5069 (11.66), +0.46%, 4/34
	3	1.75	1.7569 (5.06), +0.39%, 1/34	1.7565 (9.39), +0.37%, 2/34
D-cut	1	1.25	1.2529 (2.93), +0.23%, 0/34	1.2520 (3.18), +0.16%, 0/34
	2	1.50	1.5027 (4.35), +0.18%, 1/34	1.4993 (21.87), −0.05%, 3/34
	3	1.75	1.7535 (4.09), +0.20%, 0/34	1.7517 (2.18), +0.10%, 0/34
M-cut	1	1.25	1.2567 (5.45), +0.53%, 3/34	1.2537 (4.26), +0.29%, 2/34
	2	1.50	1.5068 (5.77), +0.46%, 4/34	1.5038 (4.67), +0.25%, 1/34
	3	1.75	1.7598 (8.73), +0.56%, 4/34	1.7572 (7.51), +0.41%, 1/34
L-cut sharp	1	1.25	1.2516 (0.83), +0.12%, 0/34	1.2509 (1.02), +0.07%, 0/34
	2	1.50	1.4820 (16.31), −1.20%, 17/34	1.4772 (15.18), −1.52%, 24/34
	3	1.75	1.6567 (12.63), −5.33%, 34/34	1.6428 (13.61), −6.12%, 34/34
L-cut rounded	1	1.25	1.2512 (0.84), +0.10%, 0/34	1.2505 (0.58), +0.04%, 0/34
	2	1.50	1.4899 (14.39), −0.68%, 10/34	1.4865 (10.37), −0.90%, 15/34
	3	1.75	1.6768 (17.17), −4.18%, 33/34	1.6541 (15.96), −5.48%, 34/34
U-cut	1	1.25	1.2282 (8.07), −1.75%, 30/34	1.2271 (6.36), −1.83%, 16/17 ^a^
	2	1.50	1.4319 (33.14), −4.54%, 33/34	1.4203 (22.81), −5.31%, 34/34
	3	1.75	1.5582 (12.64), −10.96%, 34/34	1.5425 (15.23), −11.86%, 34/34
Scan-cut	1	1.25	1.2528 (1.85), +0.23%, 0/34	1.2508 (1.22), +0.07%, 0/34
	2	1.50	1.5031 (3.03), +0.20%, 0/34	1.5025 (2.66), +0.16%, 0/34
	3	1.75	1.7548 (3.34), +0.27%, 0/34	1.7522 (3.10), +0.13%, 0/34

**Table 4:** Thermal-shock ΔR/R per panel and per row for the two laser-modification routes. ⊥, scan lines perpendicular to the current path; ‖, scan lines parallel to the current path.

Route	Panel	Row (Treatment)	*n*	Mean ΔR/R (%)	SD (%)	Max Abs. ΔR/R (%)	Within ±1.0%
Pico	#8	Row 1 (⊥ laser -modified)	10	+0.67	0.11	0.89	10/10
Pico	#8	Row 2 (untreated control)	10	+0.53	0.13	0.72	10/10
Pico	#8	Row 3 (‖ laser-modified)	10	+0.63	0.13	0.84	10/10
Pico	#9	Row 1 (⊥ laser-modified)	10	+0.67	0.10	0.81	10/10
Pico	#9	Row 2 (untreated control)	10	+0.46	0.13	0.66	10/10
Pico	#9	Row 3 (‖ laser-modified)	10	+0.62	0.12	0.79	10/10
MOPA	*e*	Row 1 (⊥ laser-modified)	10	+0.69	0.24	1.13	9/10
MOPA	*e*	Row 2 (untreated control)	10	+0.51	0.17	0.68	10/10
MOPA	*e*	Row 3 (‖ laser-modified)	10	+0.51	0.24	0.96	10/10

**Table 5:** Aggregated within-panel comparison of laser-modified rows against the same-panel untreated row.

Route Contrast (Rows 1 + 3 vs. Row 2)	*n* (mod)	Mean ΔR/R (mod,%)	*n* (ctrl)	Mean ΔR/R (ctrl, %)	Δ (Mod − Ctrl, pp)	Inference
Pico (panels #8 + #9)	40	+0.65	20	+0.50	+0.15	Descriptive only
MOPA (panel *e*)	20	+0.60	10	+0.51	+0.09	Descriptive only

**Table 6:** Production-planning summary of the two laser-modification routes on the 8P7R experimental vehicle.

Criterion	Pico Route	MOPA Route
Modification recipe (Step 1)	10–12 ps, 1064 nm, 100 kHz, 1 W, F100, area scan	≈6 ns, 1064 nm, 100 kHz, 1.4 W, F160, area scan
Resistance recovery from post-fire	39% below Ri (Rf/Ri = 0.607 ± 0.015, *n* = 3192); 3192/3192 at Rf/Ri ≤ 0.870	17% below Ri (Rf/Ri = 0.831 ± 0.052, *n* = 1175); 912/1175 (77.6%) at Rf/Ri ≤ 0.870
Overshoot events (Rf > Ri)	0/3192	0/1175
Up-trim SD of trim ratio, 7 kerfs (shared trimmer)	0.73–13.45 $\times 10^{-3}$ (Table 3)	0.68–7.57 $\times 10^{-3}$ (Table 3)
Resistors outside ±1% after up-trim (per row, two panels per route)	Scan-cut: none; I-, D-, M-cut: 0–4 of 34; L-cuts: none at +25%, 10–24 of 34 at +50%, target not reached at +75%; U-cut: 30–34 of 34, not suitable	
Up-trim equivalence (15 matched cells, both route means within ±1%)	Mean-ratio difference $+1.6\times 10^{-3}$ (90% CI +0.9 to $+2.2\times 10^{-3}$), TOST p<0.001 ($\pm 5\times 10^{-3}$); SD ratio 0.99; margin exceeded if all 21 cells are included	
Thermal-shock ΔR/R, laser-modified rows (paired)	+0.65% ± 0.11% (*n* = 40, panels #8 + #9); 40/40 within ±1%	+0.60% ± 0.25% (*n* = 20, panel *e*); 19/20 within ±1%
Thermal-shock ΔR/R, within-panel untreated row (paired)	+0.50% ± 0.13% (*n* = 20, panels #8 + #9); 20/20 within ±1%	+0.51% ± 0.17% (*n* = 10, panel *e*); 10/10 within ±1%
Laser-associated increment (modified − within-panel untreated), 5 cycles −30/+80 °C	+0.15 pp; within the ±0.5 pp engineering margin (TOST, two panels)	+0.09 pp; preliminary (one panel)
Difference in TCR stage means, post-trim to post-thermal-shock (Row 1; different resistor sets for Pico)	+12.4 ppm °C^−1^	+24.9 ppm °C^−1^
TCR post-laser (Row 1)	−47 ± 15 ppm °C^−1^ (*n* = 154)	+40 ± 18 ppm °C^−1^ (*n* = 56)
TCR post-thermal-shock (Row 1)	−23 ± 7 ppm °C^−1^	+67 ± 8 ppm °C^−1^
TCR after 155 °C × 100 h (Row 1)	−14 ± 8 ppm °C^−1^	+82 ± 19 ppm °C^−1^
TCR after 155 °C, resistors within ±100 ppm °C^−1^	Row 1: 10/10; Row 3: 10/10	Row 1: 10/10; Row 3: 8/10 (maximum +107)
SEM/EDS evidence	No macroscopic delamination; principal elemental signatures retained	No macroscopic delamination; principal elemental signatures retained
Method-validation outcome (this study)	Feasibility supported on two tested 8P7R panels; modified-minus-control ΔR/R contrast = +0.15 pp	Preliminary feasibility supported on one tested 8P7R panel; modified-minus-control ΔR/R contrast = +0.09 pp

**Table 7:** Differences between the 8P7R SIP-8 vehicle and a resistor embedded in a multilayer HIC substrate, and their implications for transfer of the present results. The entries identify re-verification needs; they are not measured results. ⊥, scan lines perpendicular to the current path; ‖, scan lines parallel to the current path.

Attribute	8P7R Vehicle (This Study)	Embedded HIC Resistor	Possible Effect and Re-Verification Need
Thermal boundary	Single printed resistor layer on a 0.635 mm 96% alumina substrate, exposed surface	Resistor surrounded by other printed layers, conductors, and possibly assembled components, with a different heat-spreading path	Heat flow during laser modification and during thermal cycling may differ, so Rf/Ri, the modified-layer state, and the thermal-shock increment must be re-measured on the HIC stack.
Substrate constraint	Single-layer film on alumina; mechanical constraint mainly from the substrate	Film constrained by overlying and underlying layers with different thermal-expansion coefficients	Thermo-mechanical stress in thermal cycling may be higher or differently distributed; longer and more severe cycling than the present five cycles is required.
Current path	Straight resistor between two terminations; scan orientation relative to the current path is well defined (Row 1 ⊥, Row 3 ‖)	Resistor geometry and terminations set by the circuit layout, possibly with bends, vias, or neighbouring conductors	The effect of scan orientation and of the up-trim kerf on the current path must be re-checked for the actual resistor geometries.
Packaging and access	SIP-8 package; resistors accessible for probing and laser processing on the open surface	Resistor may be covered or located near other structures; trim feedback must use the circuit’s test pads	Beam access, focus, and closed-loop measurement access must be re-qualified; encapsulation or coating after trimming may change the stress state.
Cost of failure	Low; an 8P7R array can be scrapped	High; a mis-processed HIC carries the full upstream cost	Qualification should proceed stepwise (dummy substrates before production HICs) with sample sizes set by the customer specification.

## Data Availability

The raw resistance, thermal-shock, and TCR datasets underlying this study are available from the corresponding author on reasonable request.

## Supplementary Materials

The following supporting information can be downloaded at: https://www.mdpi.com/article/10.3390/mi17101164/s1, Figure S1: Kerf width of the up-trim cut, measured at two sites with the trimmer inspection microscope and a two-axis FUJI linear scale: (a) site a, 0.042 mm; (b) site b, 0.043 mm (displayed scale readings).; Figure S2: Optical micrographs of the seven up-trim kerf geometries used in Table 3 and Figure 3 of the main text: (a) I-cut, (b) D-cut, (c) M-cut, (d) L-cut with sharp corner, (e) L-cut with rounded corner, (f) U-cut, (g) scan-cut. All were cut on the same shared trimmer under closed-loop resistance feedback.; Table S1: Up-trim kerf dimensions and what was, and was not, measured; Table S2: Per-panel up-trim results underlying main-text Table 3 (interior positions 3–19 of each row, n = 17 per panel row). Entries: mean trimmed ratio (SD × 103), number of resistors outside ±1% of target / n. Targets: Row 1, 1.25; Row 2, 1.50; Row 3, 1.75. ^a^ Excluded: the record contains ratios below 1 and was treated as a recording error. Generated by uptrim table.py from the per-panel sheets of the raw up-trim workbook.

## Abbreviations

The following abbreviations are used in this manuscript:

CV	Coefficient of variation
EDS	Energy-dispersive X-ray spectroscopy
EHT	Electron high tension (SEM accelerating voltage)
HIC	Hybrid integrated circuit
IEC	International Electrotechnical Commission
LTCC	Low-temperature co-fired ceramic
MOPA	Master-oscillator power-amplifier (fiber laser)
Pico	Picosecond (laser)
pp	Percentage point(s): the arithmetic difference between two percentages
Ri, Rf	As-fired resistance; resistance after surface modification
SD	Standard deviation
SDD	Silicon-drift detector
SE2	Chamber secondary-electron detector (Zeiss)
SEM	Scanning electron microscopy
SIP	Single in-line package
TCR	Temperature coefficient of resistance
TOST	Two one-sided tests (equivalence test)
XPS	X-ray photoelectron spectroscopy
XRD	X-ray diffraction
