# Supplementary material for: Recovery of Over-Target RuO2-Based Thick-Film Resistors by Laser Surface Modification and Dual-Step Trimming: An 8P7R Feasibility Study Toward Multilayer Hybrid Integrated Circuits
Source: Micromachines (Basel). 2026 Oct 5;17(10):1164. doi: 10.3390/mi17101164 (PMC13707958; doi:10.3390/mi17101164)
Supplement: Supplementary file 1 [file micromachines-17-01164-s001.zip › Supplementary Materials.pdf]

Supplementary Materials

Recovery of Over-Target RuO_2-Based Thick-Film Resistors by Laser Surface Modification and Dual-Step Trimming: An 8P7R Feasibility Study toward Multilayer Hybrid Integrated Circuits (micromachines-4576354, revised)

This supplement documents the up-trim kerfs cut on the shared laser trimmer (main text, Section 2.2). The surface-modification step (Pico or MOPA) is an area scan of the whole resistor and does not cut a kerf; the kerfs shown here are made only in the subsequent up-trim step.

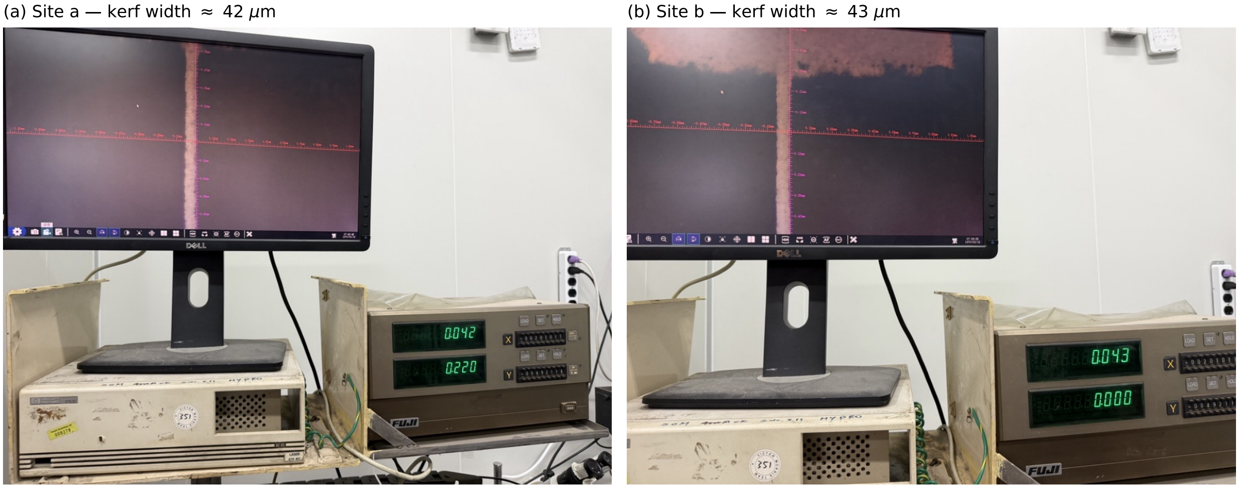

Figure S1: Kerf width of the up-trim cut, measured at two sites with the trimmer inspection microscope and a two-axis FUJI linear scale: (**a**) site a, 0.042 mm; (**b**) site b, 0.043 mm (displayed scale readings).

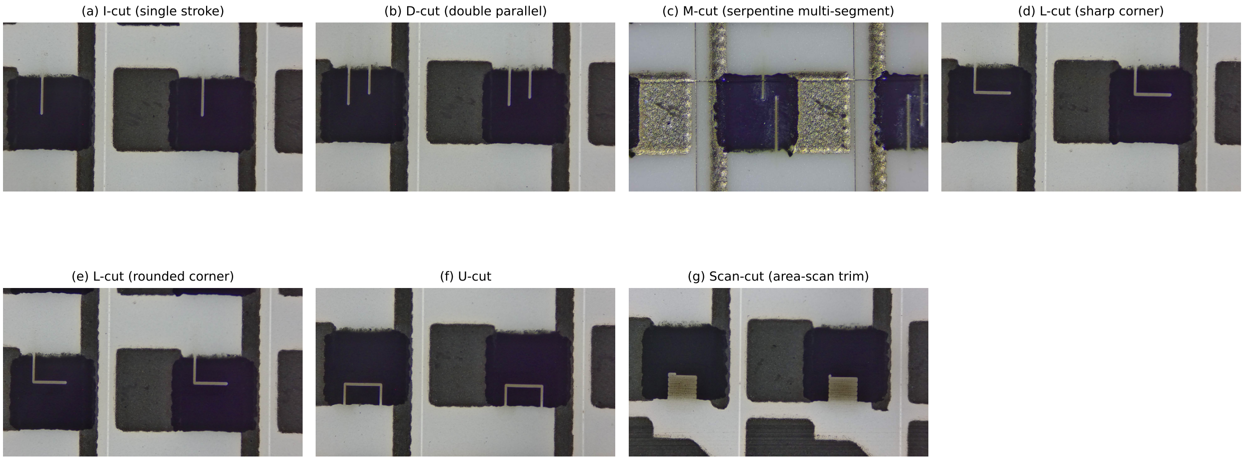

Figure S2: Optical micrographs of the seven up-trim kerf geometries used in Table 3 and Figure 3 of the main text: (**a**) I-cut, (**b**) D-cut, (**c**) M-cut, (**d**) L-cut with sharp corner, (**e**) L-cut with rounded corner, (**f**) U-cut, (**g**) scan-cut. All were cut on the same shared trimmer under closed-loop resistance feedback.

Table S1: Up-trim kerf dimensions and what was, and was not, measured.

**Parameter**	**Value**	**Method / notes**
Kerf width	0.042 and 0.043 mm	Two sites; inspection microscope + FUJI linear scale (Figure S1)
Kerf depth	Not measured. Intended: through the full printed film to the alumina substrate (nominal fired-film thickness ≈10 μm)	No cross-sectional microscopy; 10 μm is a nominal film thickness, not a measured kerf depth
Kerf length	Not fixed; not logged per resistor	Closed-loop trimming stops the cut when the target resistance is reached, so the length varies with the pre-trim resistance and target (limitation)
Trimmer	One shared laser trimmer for all panels	Independent of the Pico/MOPA modification route
Kerf geometries	I, D, M, L (sharp), L (rounded), U, scan-cut	Figure S2; results in main-text Table 3
Surface-modification step	Area scan of the whole resistor; no kerf	Pico: 10–12 ps, 100 kHz, 1 W, 2000 mm s^{-1}, 1 pass, hatch 0.01 mm, F100. MOPA: ≈6 ns, 100 kHz, 1.4 W, 2000 mm s^{-1}, 1 pass, hatch 0.01 mm, F160. Modified-layer depth not measured

Table S2: Per-panel up-trim results underlying main-text Table 3 (interior positions 3–19 of each row, $n = 17$ per panel row). Entries: mean trimmed ratio (SD×10^3), number of resistors outside ±1 % of target / n. Targets: Row 1, 1.25; Row 2, 1.50; Row 3, 1.75. [a] Excluded: the record contains ratios below 1 and was treated as a recording error. Generated by `uptrim_table.py` from the per-panel sheets of the raw up-trim workbook.

Kerf	Row	Pico #2	Pico #5	MOPA e	MOPA f
I-cut	1	1.2536 (2.10), 0/17	1.2545 (2.93), 0/17	1.2541 (2.79), 0/17	1.2523 (2.40), 0/17
	2	1.5067 (14.81), 1/17	1.5032 (3.07), 0/17	1.5069 (10.21), 2/17	1.5069 (13.27), 2/17
	3	1.7575 (3.67), 0/17	1.7562 (6.20), 1/17	1.7547 (4.93), 0/17	1.7584 (12.26), 2/17
D-cut	1	1.2536 (3.38), 0/17	1.2521 (2.27), 0/17	1.2508 (1.38), 0/17	1.2532 (3.99), 0/17
	2	1.5019 (3.43), 0/17	1.5036 (5.07), 1/17	1.4953 (30.38), 2/17	1.5032 (5.46), 1/17
	3	1.7532 (3.55), 0/17	1.7538 (4.66), 0/17	1.7515 (2.01), 0/17	1.7519 (2.39), 0/17
M-cut	1	1.2582 (7.07), 3/17	1.2551 (2.51), 0/17	1.2523 (3.31), 0/17	1.2551 (4.73), 2/17
	2	1.5071 (7.07), 3/17	1.5066 (4.29), 1/17	1.5039 (5.37), 1/17	1.5037 (4.01), 0/17
	3	1.7591 (11.71), 2/17	1.7604 (4.38), 2/17	1.7582 (9.70), 1/17	1.7563 (4.50), 0/17
L-cut sharp	1	1.2513 (0.64), 0/17	1.2519 (0.91), 0/17	1.2514 (1.09), 0/17	1.2504 (0.67), 0/17
	2	1.4886 (11.23), 6/17	1.4754 (18.16), 11/17	1.4847 (14.26), 8/17	1.4697 (12.33), 16/17
	3	1.6582 (11.68), 17/17	1.6552 (13.70), 17/17	1.6505 (12.02), 17/17	1.6352 (10.64), 17/17
L-cut rounded	1	1.2510 (0.83), 0/17	1.2514 (0.84), 0/17	1.2504 (0.67), 0/17	1.2507 (0.45), 0/17
	2	1.4953 (9.05), 2/17	1.4844 (16.78), 8/17	1.4895 (9.93), 7/17	1.4835 (10.22), 8/17
	3	1.6813 (11.40), 17/17	1.6724 (20.88), 16/17	1.6630 (14.97), 17/17	1.6451 (11.46), 17/17
U-cut	1	1.2265 (8.44), 16/17	1.2299 (7.55), 14/17	excl.[a]	1.2271 (6.36), 16/17
	2	1.4447 (33.42), 16/17	1.4191 (28.27), 17/17	1.4324 (24.05), 17/17	1.4082 (13.57), 17/17
	3	1.5607 (8.19), 17/17	1.5557 (15.79), 17/17	1.5531 (10.80), 17/17	1.5318 (10.95), 17/17
Scan-cut	1	1.2534 (1.90), 0/17	1.2523 (1.69), 0/17	1.2512 (1.47), 0/17	1.2505 (0.83), 0/17
	2	1.5025 (2.08), 0/17	1.5037 (3.72), 0/17	1.5035 (2.82), 0/17	1.5014 (2.09), 0/17
	3	1.7561 (3.46), 0/17	1.7535 (2.72), 0/17	1.7534 (3.91), 0/17	1.7510 (1.21), 0/17
